# Interplay between Structure and Charge as a Key to Allosteric Modulation of Human 20S Proteasome by the Basic Fragment of HIV-1 Tat Protein

**DOI:** 10.1371/journal.pone.0143038

**Published:** 2015-11-17

**Authors:** Przemysław Karpowicz, Paweł A. Osmulski, Julia Witkowska, Emilia Sikorska, Małgorzata Giżyńska, Agnieszka Belczyk-Ciesielska, Maria E. Gaczynska, Elżbieta Jankowska

**Affiliations:** 1 Department of Biomedical Chemistry, Faculty of Chemistry, University of Gdańsk, Gdańsk, Poland; 2 Department of Molecular Medicine, Institute of Biotechnology, University of Texas Health Science Center at San Antonio, San Antonio, Texas, United States of America; 3 Department of Organic Chemistry, Faculty of Chemistry, University of Gdańsk, Gdańsk, Poland; 4 Institute of Biochemistry and Biophysics, Polish Academy of Sciences, Warsaw, Poland; University of Cambridge, UNITED KINGDOM

## Abstract

The proteasome is a giant protease responsible for degradation of the majority of cytosolic proteins. Competitive inhibitors of the proteasome are used against aggressive blood cancers. However, broadening the use of proteasome-targeting drugs requires new mechanistic approaches to the enzyme’s inhibition. In our previous studies we described Tat1 peptide, an allosteric inhibitor of the proteasome derived from a fragment of the basic domain of HIV-Tat1 protein. Here, we attempted to dissect the structural determinants of the proteasome inhibition by Tat1. Single- and multiple- alanine walking scans were performed. Tat1 analogs with stabilized beta-turn conformation at positions 4–5 and 8–9, pointed out by the molecular dynamics modeling and the alanine scan, were synthesized. Structure of Tat1 analogs were analyzed by circular dichroism, Fourier transform infrared and nuclear magnetic resonance spectroscopy studies, supplemented by molecular dynamics simulations. Biological activity tests and structural studies revealed that high flexibility and exposed positive charge are hallmarks of Tat1 peptide. Interestingly, stabilization of a beta-turn at the 8–9 position was necessary to significantly improve the inhibitory potency.

## Introduction

The 26S proteasome, a main component of the ubiquitin-proteasome proteolytic system, consists of the 20S catalytic core and two 19S regulatory particles (RP) attached to both sides of the core. The 19S RP is responsible for recognition and binding of polyubiquitinylated protein substrates, their deubiquitinylation, unfolding, and translocation to the catalytic chamber [1]. Two other proteasome activators - 11S (PA28/REG) and PA200 do not process polyubiquitinylated proteins. The PA28 αβ/REG/11S activator enhances proteolysis of peptides and unstructured proteins and its physiological role is to activate production of antigenic peptides [2]. PA200 is predominantly found in PA200-20S-19S complexes, which are involved mainly in DNA repair and maintaining mitochondrial function [3].

The 20S core is built with four stacked heptameric rings arranged in the *αββα* order [4]. The inner *β*-rings harbor six active centers with three distinct activities: post-glutamyl peptide hydrolyzing (PGPH), trypsin-like (T-L) and chymotrypsin-like (ChT-L), located in β1, β2 and β5 subunits, respectively. An access of protein substrates to the catalytic chamber is restricted by the tightly packed *N*-terminal tails of the *α* subunits, forming the regulated gate [5], which can be opened by binding the 11S, 19S or PA200 activators. It is postulated that all the activating particles display a common mechanism of action, involving anchoring through their C-terminal residues to the proteasome surface, and opening the gate by repositioning the Pro17 reverse turn in one or multiple α-subunits [6]. The activators do not only force gate opening, but also influence the proteasome catalytic performance by allosteric signalling [7]. Importantly, allosteric signalling plays a critical yet not fully understood role in the functioning of the 20S core and its interactions with ligands [8–10].

The ubiquitin-proteasome system is involved in degradation of a variety of proteins, regulating either directly or indirectly many cellular processes, such as signal transduction, metabolism, cell cycle, and apoptosis [11]. This broad influence on physiological processes makes the proteasome an important target for drugs [12], especially anti-cancer [13,14]. Many competitive active sites-directed (orthosteric) inhibitors of the proteasome have been developed. Two of them, bortezomib and carfilzomib, are already used in the treatment of blood cancers, and a few others are in clinical trials [15,16]. Unfortunately, a significant number of patients do not respond to these drugs or develop the drug resistance [17]. Allosteric modulators provide a promising alternative to the orthosteric competitive inhibitors. Allosteric regulation of an enzyme or receptor activity involves binding of an effector to a site distinct from the active site, modifying its affinity toward natural substrates through long-distance conformational changes [18]. Allosteric modulators may serve as exceptionally precise tools, since they bind to very unique sites that are generally independent from the main function of the protein. If allosteric binding sites are not already involved in regulation of the target protein they are likely under lower evolutionary pressure than catalytic sites [19,20]. Often allosteric modulators may distinguish between protein targets belonging to the same family and possessing similar active sites, what in turn allows for avoiding many side effects typical for the therapy with orthosteric modulators. It is thus not surprising there is an increasing interest in utilizing the allosteric modulation phenomena in drug design [21–23].

We postulate that allosteric modulators of the proteasome may improve efficacy of treatment, limit off-target effects, and help to overcome resistance to competitive drugs. Recently, several allosteric inhibitors of the proteasome have been identified, including proline and arginine rich peptides [24], compounds with imidazoline scaffolds [25,26], chloroquine [27], clioquinol [28], rapamycin [29] and 5-amino-8-hydroxyquinoline, some of them overcoming certain forms of bortezomib resistance *in vitro* [26,30]. In our search for allosteric modulators of the proteasome proteolytic activity, we focused on fragments of Tat (transactivator) protein from human immunodeficiency virus type-1. This poorly structured 86-residue, RNA-binding protein regulates expression of viral and host genes, including genes encoding immunoproteasome subunits, in virus-infected human cells [31]. It was observed that, besides its canonical function, the HIV-1 Tat protein competes with the 11S activator and also inhibits the core 20S proteasome [32]. These actions are a part of the broad assault of the virus on cellular immune response [31]. The protein was found to bind to several single, separately expressed α and β subunits [33], however the binding was likely encouraged by the lack of whole-assembly context. To the contrary, the competition with PA28/REG activator is likely a result of much more specific interactions of HIV-1 Tat with the α-face of the core proteasome. Interestingly, the 11S activator shares the RTP (REG/Tat proteasome binding) motif with the viral protein [34]. The RTP site is located near the C-terminal region and activation loop of the PA28/REG and in the essential basic domain of HIV-1 Tat [35]. In our previous studies we concentrated on these specific interactions and designed short, twelve-residue peptides–fragments of the basic domain containing the RTP site [36,37] We found that the peptide Tat1 (G^48^RKKRRQRRRPS^59^), inhibits the ChT-L peptidase of the activated 20S proteasome in a noncompetitive manner [36]. The result, together with competition of the peptide with 11S activator [36], strongly implies that Tat1 does not bind to the enzyme active sites, but instead it interferes with binding of the 11S activator. This suggests that the binding site for the peptide resides on the α face, within the proteasome α-subunits. Moreover, the Tat1 peptide is most likely an allosteric inhibitor: it affects the three proteasome peptidase activities in a very distinct manner [36]. This excludes the notion that the Tat1 actions stem from a simple blocking of the proteasome gate and attenuating the flow of substrates. Interestingly, in preliminary experiments we found that treatment with Tat-derived peptides, including Tat1, does affect cell viability and activities of proteasome assemblies in cultured cells (data not shown). To optimize the structure of Tat1-based modulators, we attempted to identify residues that are crucial for the peptide activity. To define the pharmacophores we performed single and multiple alanine scans and tested activity of the 20S proteasome treated with the modified peptides. We also characterized the structure of the peptides with Fourier transform infrared (FTIR) and circular dichroism (CD) spectroscopy. As a result, we identified two “hot spots” in the sequence of Tat1, comprising the residues 4–6 and 8–10. According to the molecular modeling (MD) these regions are prone to the formation of a structural turn [36]. Therefore, we incorporated a potential β-turn-inducer, the Tic-Oic moiety, to these regions to obtain peptidomimetics with a stabilized secondary structure. Next, we characterized these peptidomimetics with CD, FTIR and nuclear magnetic resonance (NMR) spectroscopy, and with molecular modeling. Propensity of certain analogs to bind the proteasome was studied with microscale thermophoresis. Biological and structural properties of the peptidomimetics indicate that although a strong positive charge is important for their inhibitory potency, a proper spatial orientation of the charged amino acid side chains is no less significant.

## Results

### Ala substitutions decreased Tat1 inhibitory potency of Tat1

To identify a pharmacophore responsible for the biological activity of Tat1 we systematically replaced selected residues in the Tat1 sequence to alanine (Table 1, S1 Table).

**Table 1:** The Ala-scan analogs of Tat1 diversely affect the ChT-L peptidase of the 20S proteasome.

Compound	Sequence	IC_50_ [μM]
Tat1	H-GRKKRRQRRRPS-OH	0.26 (±0.00)
Tat1_A3-4	H-GR**AA**RRQRRRPS-OH	0.24 (±0.02)
Tat1_A4-5	H-GRK**AA**RQRRRPS-OH	0.26 (±0.03)
Tat1_A5-6	H-GRKK**AA**QRRRPS-OH	0.32 (±0.02)
Tat1_A2-4	H-G**AAA**RRQRRRPS-OH	0.45 (±0.02)
Tat1_A3-5	H-GR**AAA**RQRRRPS-OH	1.57 (±0.22)
Tat1_A4-6	H-GRK**AAA**QRRRPS-OH	1.72 (±0.02)
Tat1_A5-7	H-GRKK**AAA**RRRPS-OH	0.31 (± 0.00)
Tat1_A5,7–9	H-GRKK**A**R**AAA**RPS-OH	1.53 (±0.14)
Tat1_A6-8	H-GRKKR**AAA**RRPS-OH	0.34 (±0.01)
Tat1_A7-9	H-GRKKRR**AAA**RPS-OH	0.28 (±0.01)
Tat1_A8-10	H-GRKKRRQ**AAA**PS-OH	2.52 (±0.03)
Tat1_A7-10	H-GRKKRR**AAAA**PS-OH	2.79 (±0.22)
Tat1_A3-6	H-GR**AAAA**QRRRPS-OH	4.24 (±0.26)
Tat1_A4-5,8–9	H-GRK**AA**RQ**AA**RPS-OH	4.66 (±0.32)
Tat1_4-5TO	H-GRK-***d*-TicOic**RQRRRPS-OH	0.24 (±0.02)
Tat1_8-9TO	H-GRKKRRQ-***d***-**TicOic**RPS-OH	0.17 (±0.03)
Tat1_8-9TOD	H-GRKKRRQ**Tic-*d*-Oic**RPS-OH	0.12 (±0.01)
Tat1_4-5TO,8-9TOD	H-GRK-***d***-**TicOic**RQ**Tic-*d*-Oic**RPS-OH	0.24 (±0.05)

An initial single-Ala walking scan did not identify residues that influenced the ability of Tat1 to inhibit the ChT-L peptidase of the 20S core proteasome (CP). Indeed, the activity of CP treated with 1 μM of these peptides varied by no more than 4% comparing to Tat1 (S1 Fig). Therefore, in the next step we proceeded with multiple Ala walking scans by exchanging blocks of 2–4 adjacent amino acid residues into alanines.

Values of half maximal inhibitory concentration (IC_50_) calculated for these analogs are presented in Table 1. Substitution of two adjacent residues (Tat1_A3-4, Tat1_A4-5, and Tat1_A5-6) had a weak (Tat1_A5-6) or insignificant influence on the inhibitory potency of the peptides. Similarly, the peptides in which three consecutive residues were exchanged but only two of them were basic (Tat1_A5-7, Tat1_A6-8 and Tat1_A7-9), displayed activity comparable to Tat1. However, the importance of charge became apparent when three basic residues were replaced with hydrophobic Ala. Such peptides (Tat1_A2-4, Tat1_A3-5, Tat1_A4-6, Tat1_A8-10) inhibited the 20S proteasome less efficiently than Tat1. Furthermore, the effect depended on the position of the exchanged residues. The most pronounced response to the basic residues substitution was observed for Tat1_A8-10, for which IC_50_ increased almost 10 times. In contrast, substitutions of residues closer to the N-terminus showed a limited influence on the ChT-L activity. The substitution of residues had the least significant effect in the case of Tat1_A2-4, for which IC_50_ was only about two times higher than for Tat1. The essential role of the C-terminal basic residues for the peptides inhibitory potency was further demonstrated with Tat1_A5,7–9 and Tat1_A7-10. In both peptides Ala replaced four residues where three were basic. Despite the identical number of the exchanged residues, the peptide which lost three positively charged residues in the 8–10 positions was a weaker inhibitor of ChT-L peptidase (IC_50_ = 2.79 μM) than the compound, in which two basic residues from the C-terminus and one in the position 5 were replaced by Ala (IC_50_ = 1.53 μM). Analysis of biological activity of Tat1 analogs with Ala-substitutions in the central part of the sequence suggested importance of yet another region in the efficient inhibition of the 20S proteasome. Among the peptides in which three Arg/Lys residues between position 3 and 8 were replaced by Ala, the Tat1_A4-6 was the poorest inhibitor. Furthermore, removal of four basic residues from the position 3 to 6 resulted in the loss of inhibitory potency of the Tat1_A3-6, further confirming importance of this region (Table 1).

### Incorporation of a Tic-Oic moiety into the pharmacophore sites specifically improves inhibitory potential of Tat1

Interestingly, the central and close to the C-terminus regions identified by the multiple-alanine walking scan were also recognized in our previous study [36]. Namely, results of molecular dynamics modeling suggested that the regions comprising residues 4–5 and 8–9 have a high propensity to form structural turns. To test whether the turns influence Tat1 activity, we introduced a dipeptide mimetic Tic-Oic (Tic: 1,2,3,4-tetrahydroisoquinoline-3-carboxylic acid, Oic: (2S,3aS,7aS)-octahydro-1*H*-indole-2-carboxylic acid) into the two postulated pharmacophore sites (Table 1), aiming to induce a stabilized β-turn conformation [38]. To check how stereochemical properties influence biological activity, we utilized both *d*- and *l*- Tic and Oic units in two combinations: *d*-Tic,*l*-Oic, incorporated to the peptides Tat1_4-5TO and Tat1_8-9TO, and *l*-Tic,*d*-Oic utilized in the peptides Tat1_8-9TOD and Tat1_4-5TO,8-9TOD. We assumed that the turn structures induced by *d*-Tic,*l*-Oic and *l*-Tic,*d*-Oic would trigger a distinct spatial arrangement of the side chains, what might impact biological activity of the peptides.

**Fig 1:**
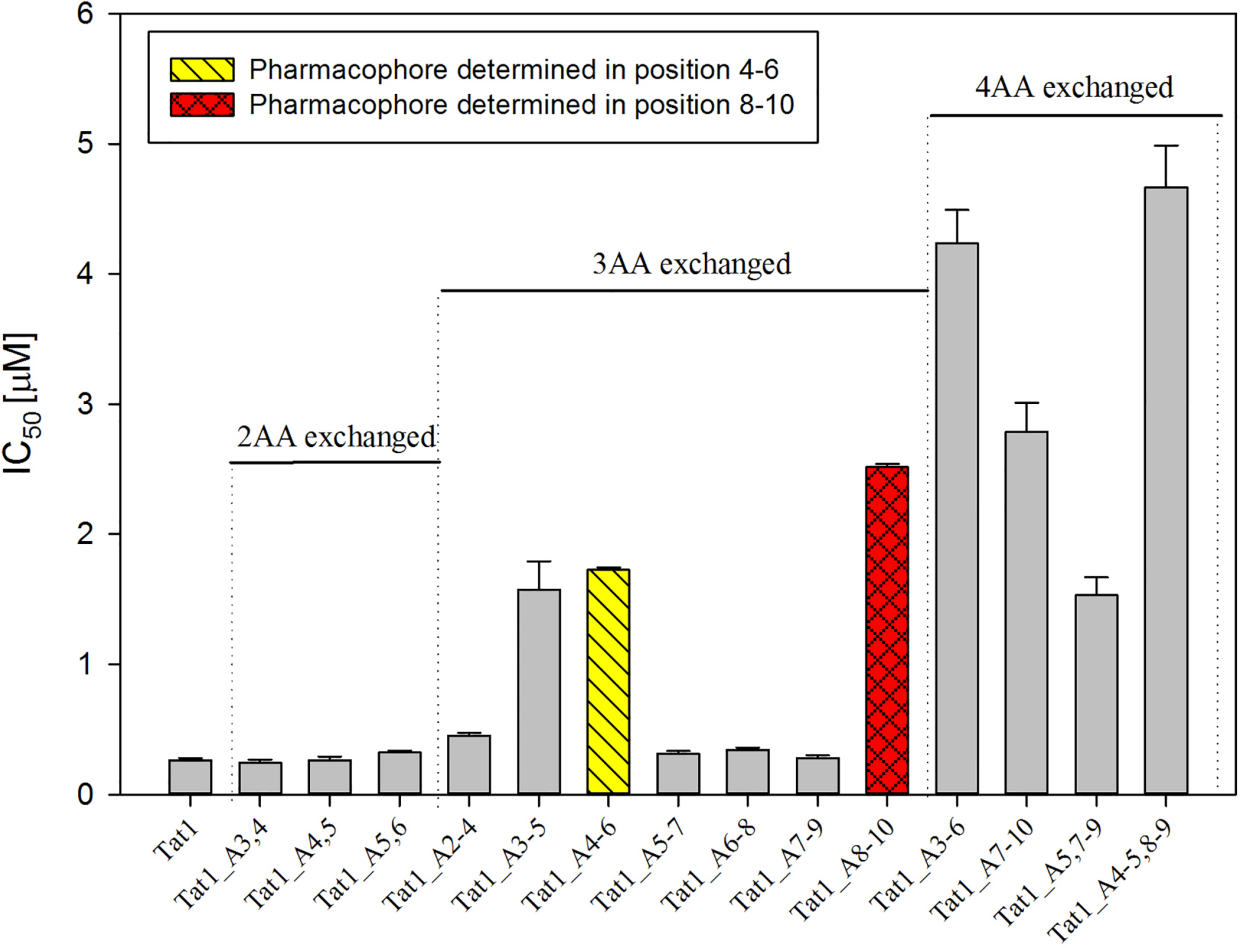
Inhibitory activity of the peptides with “pharmacophore” regions modified by the Tic-Oic moiety.

Due to the Tic-Oic modification the overall positive charge of the peptides decreased because Tic-Oic moiety substituted either two (Tat1_4-5TO, Tat1_8-9TO, Tat1_8-9TOD) or four Arg/Lys residues (Tat1_4-5TO,8-9TOD). As a control we prepared Tat1_A4-5,8–9 peptide, in which four Ala residues were introduced in the relevant regions. Comparison of biological effectiveness of the obtained compounds indicated that their inhibitory capacity is not purely dependent on charge. While the peptide with four basic residues replaced by Ala had drastically reduced potency to inhibit the 20S proteasome (Table 1), Tat1_4-5TO, Tat1_8-9TO/TOD and Tat1_4-5TO,8-9TOD inhibited the 20S core with at least comparable effectiveness as Tat1 (Fig 1). Importantly, Tat1_8-9TO and especially Tat1_8-9TOD inhibited the 20S proteasome significantly better than Tat1, with IC_50_ reduced by half.

It thus seems that incorporation of the Tic-Oic moiety as a β-turn inducer leads to a locally more rigid peptide structure that in the certain context supports a better fit to proteasome binding domain(s) and consequently stimulates more efficient propagation of allosteric signals. Moreover, *l*-Tic,*d*-Oic at the position 8–9 provides a better fitting of the ligand since IC_50_ for Tat1_8-9TOD is about 30% lower than for its counterpart with *d*-Tic,*l*-Oic unit.

### The peptide with Ala substitutions in both pharmacophore regions forms the least stable complex with the proteasome

Microscale thermophoresis detects changes in the mobility of protein molecules in temperature gradients generated by IR laser radiation. The thermophoretic effect is very sensitive to induced by a ligand binding alterations in the charge, size and/or the hydration shell of a studied protein [39]. Quantification of these changes is based on either intrinsic fluorophores such as tryptophan or covalently bound dyes. Titration of the selected peptides/peptidomimetics to a constant concentration of the fluorescently labeled human 20S proteasome allowed us to determine ligand dissociation constants (K_d_), and systematically compare their binding affinity. The calculated K_d_ values (S2 Table) were substantially higher than those expected based on the corresponding IC_50_, which most probably were the result of the introduced labeling, likely decreasing the affinity of the peptides to the 20S proteasome. Moreover, the labelling also decreased the peptidase activity of the proteasome. Despite these limitations, the binding tendency of the studied Tat1 analogs followed their inhibitory capacity (S2 Fig). For example, Tat1_A4-5,8–9, the worst 20S inhibitor in this set of modulators, was found as forming the least stable complex with the proteasome (K_d_ = 21.40 μM). The equivalent peptidomimetic with Tic-Oic units simultaneously incorporated into the 4–5 and 8–9 positions was characterized by a much lower dissociation constant (K_d_ = 8.38 μM) indicating stronger interaction. Importantly, a comparison of the K_d_ values for Tat1_8-9TO and Tat1_8-9TOD confirmed a better fitting of the *l*-Tic,*d*-Oic than *d*-Tic,*l*-Oic moiety to the proteasome surface, as was suggested on the basis of the biological activity results.

### FTIR determined conformation of Tat1 analogs depends on their structural flexibility

The amide I band (1600–1700 cm^-1^), that represents predominantly the stretching vibration of the amide C = O group, usually serves as a structural probe in FTIR of peptides and proteins [40]. Since the strength of C = O bond depends greatly on its hydrogen bonds network, various elements of secondary structure give rise to different but strongly overlapping signature bands that form an amide I envelope. These bands can be extracted from the envelope by carrying out the second derivative transformation or deconvolution of the spectra [41,42].

FTIR spectra of the parental Tat1 peptide were recorded either in a form of an aqueous solution or as a film prepared by solvent evaporation from the samples spotted on a FTIR window. The latter approach allowed elimination of subtracting a water background though not interfering with the band deconvolution. We applied this method since it was reported (and confirmed by us [43]) that evaporation of a solvent in most cases preserves the conformation [44]. Apparently, short linear peptides such as Tat1 and its analogs did not follow this rule, since the spectra of the peptides in solution and in the form of a film were distinct.

**Fig 2:**
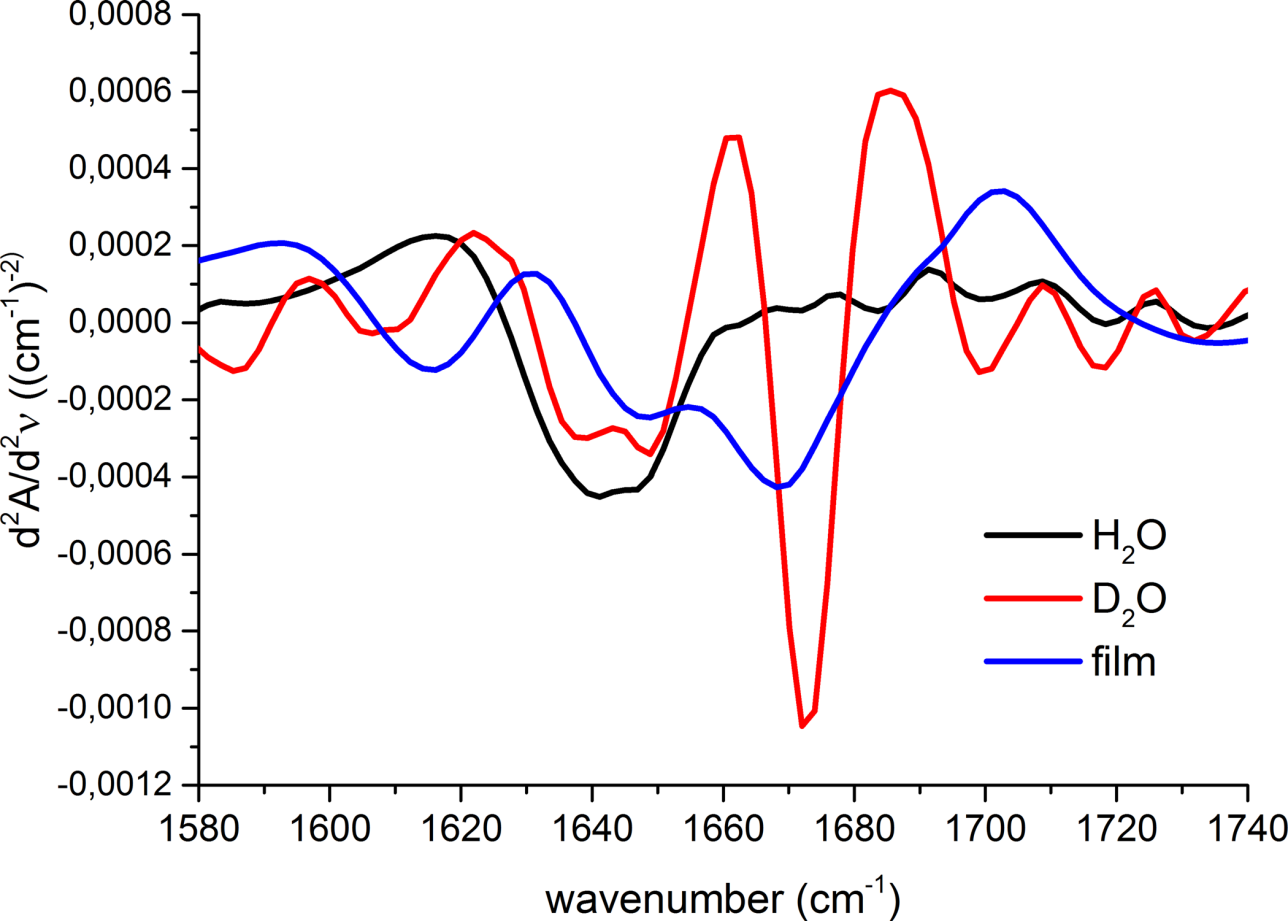
The second derivative of FTIR spectra of Tat1, recorded in H_2_O, D_2_O and in a film mode.

For Tat1 peptide in water, a single broad minimum extending between 1630 and 1655 cm^-1^ was detected (Fig 2). This minimum was probably formed by a combination of at least two bands centered at about 1638 and 1645 cm^-1^, which can be assigned to a β-sheet and a random coil conformation, respectively [45]. The β-sheet conformation was further confirmed by presence of another band typical for an antiparallel β-sheet, above 1690 cm^-1^. The other, very weak, minima localized at about 1670 and above 1680 cm^-1^ can be ascribed to turns.

In contrast, Tat1 in the form of a film produced three separate minima–at about 1615, 1645, and 1670 cm^-1^, which were attributed to the polyproline II (PPII), random coil, and turn conformations, respectively [36,46] (Fig 2). Among them, the minimum located at 1670 cm^-1^ was the most pronounced suggesting that the structural constrains imposed by the film increased preferences for the turn conformation. Surprisingly, this minimum also appeared as the main feature of Tat1 spectrum recorded in D_2_O (Fig 2). Preservation of this minimum implicates that although the bent structure of Tat1 was not stabilized by O^…^H and/or N^…^H hydrogen bonds strongly enough to be a preferred conformation in aqueous solution, the O^…^D and/or N^…^D bonds were sufficiently stronger to support the turn.

All the Ala-scan peptides in an aqueous solution consistently produced a broad minimum with the center between 1640 and 1650 cm^-1^ (S3 Fig), representing an unordered conformation. Spectra of these peptides in the form of a film displayed two minima. The first broad minimum extended between 1625 and 1650 cm^-1^ indicating an unordered conformation with some preferences for a β-sheet. The second and dominating minimum at about 1680 cm^-1^ was assigned to turns. For the selected peptides we also collected spectra in D_2_O. Similarly to the response of Tat1 to heavy water, these peptides adopted a more ordered conformation reflected by predominance of the minimum at 1670 cm^-1^ that is a hallmark of a turn structure (S4 Fig).

### CD confirms propensity of the Ala-scan peptides for adopting an extended conformation

A CD spectrum of Tat1 displayed a weak maximum around 220 nm and a deep minimum at 195–198 nm [36], which together constitute a signature of an unordered or polyproline II (PPII) conformation [47,48]. The previously observed temperature-dependent changes in the spectrum of Tat1 strongly support the latter assignment [36]. The recorded CD spectra for Tat1 analogs, with both the single and multiple Ala substitutions, in general follow the pattern observed for the parental Tat1, indicating the Ala scan peptides also adopt an extended PPII conformation (S5 Fig).

### A β-turn is present in the peptides with the TOD unit in the position 8–9

**Fig 3:**
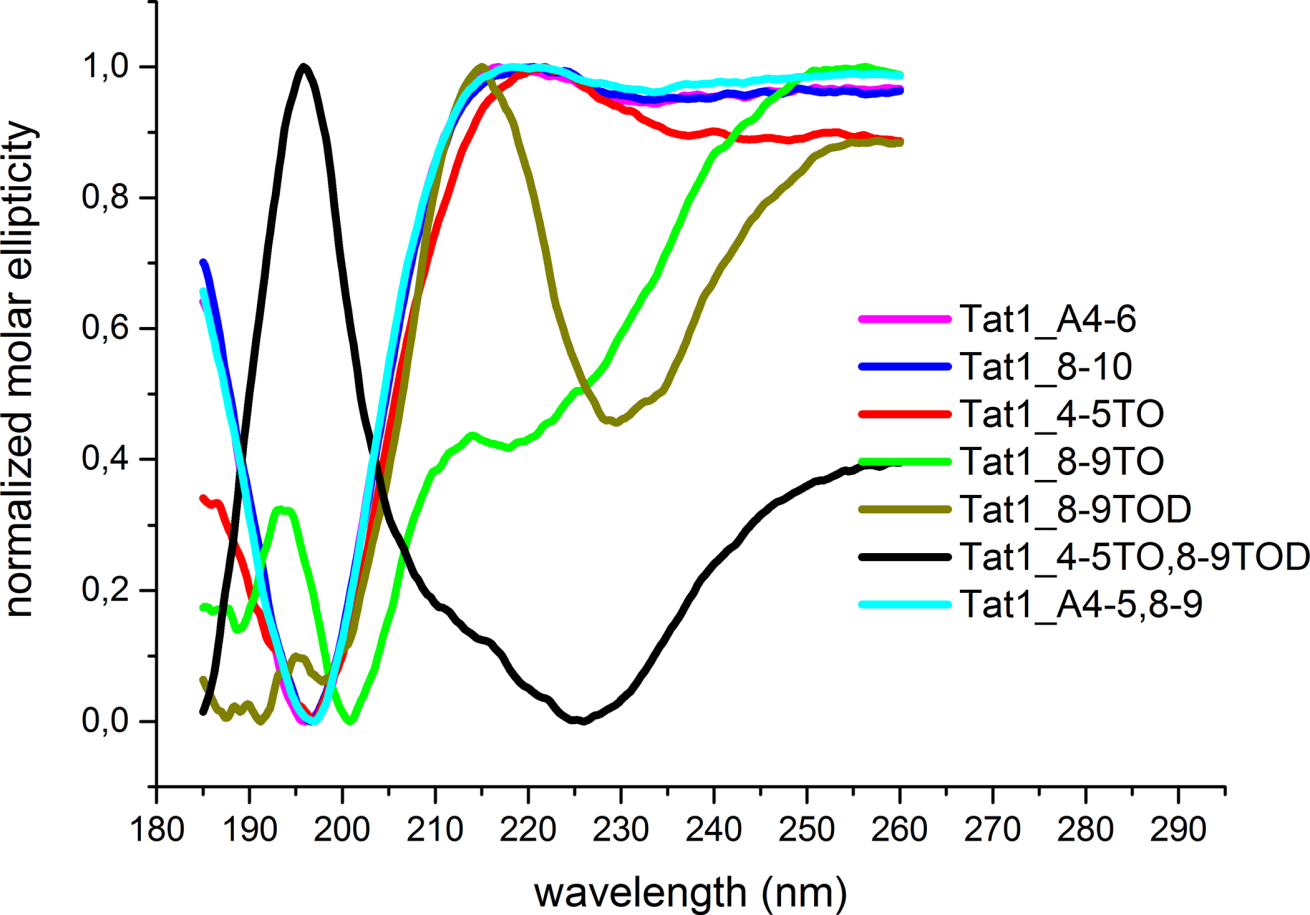
Normalized CD spectra of Tat1 analogs in which the pharmacophore residues were substituted by either Ala or Tic-Oic moiety.

More variations appeared in CD spectra of Tat1 analogs with the Tic-Oic moiety. Tat1_4-5TO peptide displayed a CD spectrum similar to Tat1 and its Ala-scan analogs, the most probably assuming the PPII conformation (Fig 3). In contrast, CD spectra of Tat1-8-9TOD and Tat1_4-5TO,8-9TOD peptides indicated that the incorporation of the *l*-Tic,*d*-Oic moiety in the position 8–9 induced a stable turn, likely of the β-type (Fig 3).

Turns are generally aperiodic secondary structures that are defined by four successive backbone torsional angles. Since the average dihedral angle values may vary at least by ±10° due to the flexibility of the structure, the turn character can be manifested in many different substructures. Using a semi-empirical approach, Woody distinguished four patterns of CD spectra characteristic for the β-turn motifs [49]. Tat1_4-5TO,8-9TOD peptide displayed a typical class C spectrum, since it showed an intensive maximum at about 195 nm and a broad α-helix-like minimum between 210 and 230 nm. This type of a spectrum was indicative of the βI conformation [50] (Fig 3). In turn, a CD spectrum of the Tat1_8-9TOD peptide, showing a minimum around 190 nm and a maximum at 215 nm, belonged to a class B spectrum, diagnostic for the βII conformation [50]. Finally, the CD spectrum of Tat1_8-9TO suggested a random coil conformation. It thus seems that *l*-Tic,*d*-Oic incorporated in the 8–9 region stabilizes the conformation better than its *d*-Tic,*l*-Oic counterpart.

### Changes in chemical shifts indicate structural rearrangements imposed by the Tic-Oic moiety

**Fig 4:**
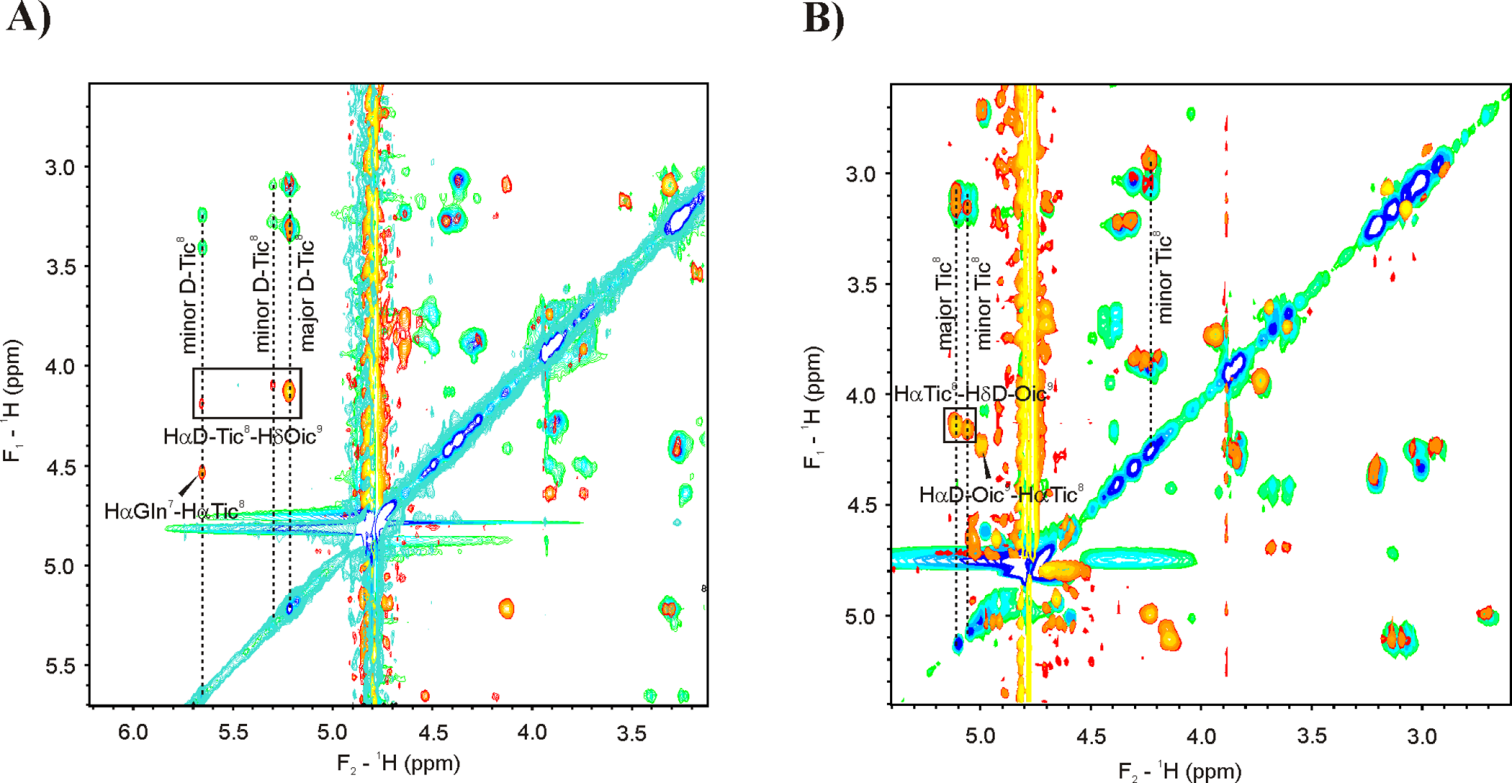
Overlaid fragments of TOCSY (green-blue) and ROESY (red-yellow) spectra of Tat1_8-9TO (A) and Tat1_8-9TOD (B).

A *cis*-*trans* interconversion of the amide bond leads in some cases to two or more sets of resonances observed in NMR spectra [51]. Indeed, a proline residue present in the sequence of Tat1 induced the NMR detectable *cis/trans* isomerization [52–55]. Due to additional tertiary amide groups present in the TO/TOD analogs of Tat1, their spectra show distinct sets of proton resonances connected with *cis/trans* isomerization of not only Arg-Pro but also Xaa-Tic and Tic-Oic bonds (S3–S7 Tables). For each Tic-Oic modified peptide the abundance of major isomer was estimated based on the intensity of the non-overlapping signals of HN-Hα of the Gln^7^ residue of each isomer observed in the TOCSY spectrum. The content of the major conformation was usually in the range of 50–80%, the highest content was observed for the analog with Tic-Oic moiety in the positions 4–5. A poor separation of signals of the major and minor conformers, or low intensities of the latter, made a complete signal assignment for the minor isomers unreliable. Thus, the full assignment was done only for the major conformer of each peptide. The sequential connectivities d_αδ_(*i*,*i+1*) found in the Tic-Oic and Arg-Pro fragments indicated presence of *trans* peptide bonds. Additionally, the chemical shift dispersion of ^13^Cβ and ^13^Cγ (Δδ_Cβ-Cγ_) in Pro residue, which falls in the range 4.7–5.1 ppm, confirmed the presence of the *trans* Arg-Pro peptide bond in the major conformations of all the peptides studied. The d_αδ_(*d*-Tic,Oic) cross peak was also found for the minor conformers of Tat1_4-5TO and Tat1_8-9TO analogs, indicating *trans* geometry of the Tic-Oic bond also in these isomers. In contrast, for Tat1_8-9TOD and Tat1_4-5TO,8-9TOD, the d_αα_(Tic,*d*-Oic) connectivity observed for one of the minor resonances evinces *cis* Tic^8^-*d*-Oic^9^ peptide bond (Fig 4). The absence of the sequential connectivities in the X-Tic dipeptide unit, i.e. d_αδ_(*i*,*i+1*) or d_αα_(*i*,*i+1*), precluded unambiguous determination of the peptide bond geometry even for the major species. However, in the ROESY spectrum of Tat1_8-9TO, the d_αα_(7,8) cross peak found for one of the minor isomers indicated a *cis* Gln^7^-Tic^8^ peptide bond (Fig 4).

**Fig 5:**
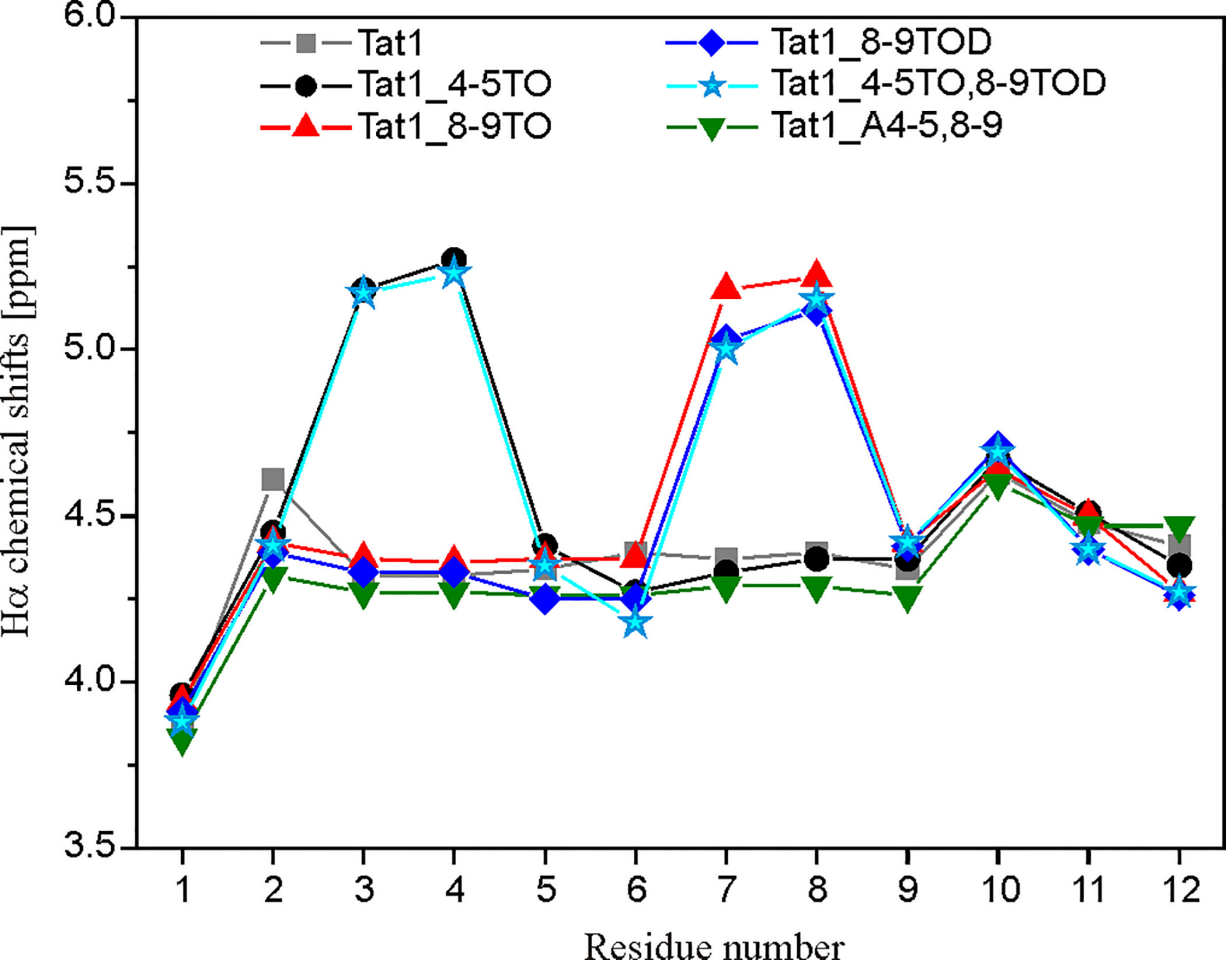
Chemical shift variation plot between Hα resonances of major conformations of Tat1, Tat1_4-5TO, Tat1_8-9TO, Tat1_8-9TOD, Tat1_4,5TO,8-9TOD and Tat1_A4-5,8–9.

Incorporation of Ala residue in positions 4, 5, 8 and 9 of Tat1 did not affect the Hα chemical shifts comparing to Tat1 [36]. In turn, in the NMR spectra of the peptides modified with Tic-Oic unit a large downfield shift of the Hα of the amino acid preceding Tic was observed. The chemical shift variation was more than 0.8 ppm for both Lys^3^ (Tat1_4-5TO and Tat1_4-5TO,8-9TOD) and Gln^7^ (Tat1_8-9TO, Tat1_8-9TOD and Tat1_4-5TO,8-9TOD) (Fig 5). These variations are potential indicators of conformational changes imposed by the TO/TOD replacement. On the other hand, by analogy to Pro [56,57], Tic and Oic are expected to have extensive effects on Hα chemical shift of the preceding residues due to the reduced conformational degrees of freedom associated with the cyclic nature of this amino acids.

The narrow HN chemical shift range (8.7–7.8 pm) found for the major conformation of each peptide was consistent with a solvent-exposed NH group that was not involved in hydrogen bonding [58]. Besides, no measurable ROEs were observed between any pair of the successive backbone amide groups (S6 Fig), which rather excluded a folded conformation. Nevertheless, the strong d_αN_(*i*,*i+1*) and d_αδ_(*i*,*i+1*) ROE cross-peaks indicated a short distance between these protons, which were expected in the ψ region of ~120±30° [59].

All the vicinal coupling constants, ^3^J_HNHα_, fell in the range of 6.5–9.3 Hz (S3–S7 Tables). Values in the range of 6.5–8 Hz are usually considered to be consistent with conformational averaging, whereas those greater than 8 Hz correspond to an extended state [51,60]. However, when the X-Pro and Pro-X pairs are in the β conformation, proline restricts conformational freedom of both the preceding and following it residues. Besides, there is a significant tendency of the residues which flank Pro to adopt the β_P_ conformation, like the proline itself [59,61]. The same behavior should be expected for the proline-like Tic and Oic residues. Thus, in the case of TO/TOD analogs of Tat1, the ^3^J_HNHα_ coupling constant values may suggest a backbone φ angle values of about -80°±20°, typical for a polyproline conformation [59].

### Conformational ensemble obtained by MD with Time-Averaged Distance Restraints

**Fig 6:**
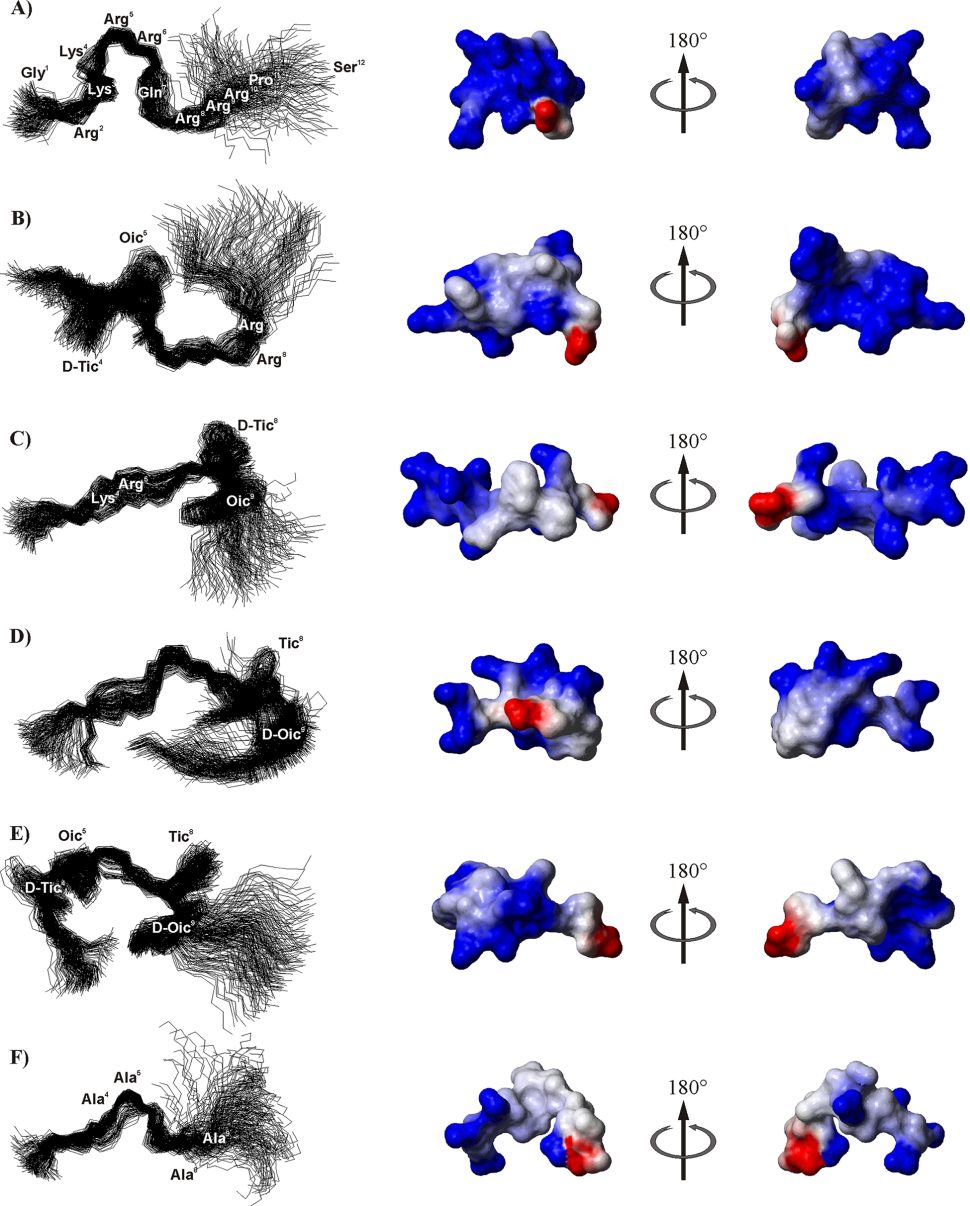
Superposed conformations obtained in the last 800 ps of MD simulations and surface electrostatic potential maps. The maps indicate regions of negative potential in red, positive potential in blue, and neutral potential in white. The figures were generated by the MOLMOL). (**A**) Tat1 [36], (**B**) Tat1_4-5TO, (**C**) Tat1_8-9TO, (**D**) Tat1_8-9TOD, (**E**) Tat1_4-5TO,8-9TOD and (**F**) Tat1_A4-5,8–9. RMSD values are 0.800, 1.008, 1.357, 1.187, 0.865 and 1.140 Å for the backbone atoms in the fragment 1–9 of Tat1, Tat1_4-5TO, Tat1_8-9TO, Tat1_8-9TOD, Tat1_4-5TO,8-9TOD and Tat1_A4-5,8–9, respectively.

Because our attempts to determine a secondary structure of the selected Tat1 analogs using ROE patterns and ^3^J_HNHα_ scalar coupling constants did not give conclusive results, especially in regard to minor conformations, molecular dynamics simulations were performed. Due to overlapping signals in the NMR spectra, the structures of Tat1_4-5TO, Tat1_8-9TO, Tat1_8-9TOD, Tat1_4,5TO,8-9TOD, and Tat1_A4-5,8–9 were solved on the basis of only small number of NMR distance restraints (Table 2). The structures obtained in the last 800 ps of MD simulations with the time-averaged distance and dihedral angle restraints are displayed in Fig 6.

**Table 2:** Statistics of distance and dihedral angle restraints used for 3D structure calculations and quality ensemble of 200 NMR-derived structures of the peptides on the last steps of MD simulations with the time-averaged distance and dihedral angle restraints.

		Peptide				
		Tat1_4-5TO	Tat1_8-9TO	Tat1_8-9TOD	Tat1_4-5TO,8-9TOD	Tat1_A4-5,8–9
**Interproton distances:**	total number	57	36	48	48	71
	intra-residue	46	27	39	37	59
	i, i+1	11	9	9	11	11
	i, i+2	0	0	0	0	1
**Dihedral angle restraints** ^**a**^		18	22	23	17	25
**Structure** ^**b**^		8,9 β I or IV (38%)	-	-	6 γ(63%)	5,6 β I or IV (81%)
**Hydrogen bonds** ^**c**^		s.c.^6^-CO^5^	-	Hε^10^-CO^8^	HN^7^-CO^5^	s.c^10^-CO^12^
**RMSD**_**1-9bb**_ **[Å]**		1.008	1.357	1.187	0.865	1.140
**Rg**_**heavy**_ **[Å]**		8.4	9.7	7.7	8.3	9.5

^a^The distance constraints and the ^3^J_HNHα_ vicinal coupling constants were used in the HABAS algorithm of the DYANA package to generate φ, ψ and χ^1^ dihedral angle restraints.

^b^ The content of the conformers with indicated turn structure is given in parentheses.

^c^ Hydrogen bonds appearing in more than 50% of the structures in the final ensemble.

Collective scatter plots of the conformational states of each amino acid residue (S7 Fig) showed a high propensity of the peptides to adopt an extended (β-strand and/or PPII) conformation. The presence of Oic residue stabilizes locally the peptide conformation by fixing its φ and ψ dihedral angles to about -60° and 150°, respectively, similarly as proline [60,61]. In Tat1_4-5TO,8-9TOD, the change of Oic^9^ configuration from *L* into *D* resulted in the “mirror image” (φ,ψ) angles, i.e. 60° and -150°, respectively. In turn, in the case of Tat1_8-9TOD, the ψ dihedral angle of *d*-Oic^9^ was able to populate the left-handed α-helical region (ψ ≈ 40°) of the Ramachandran plot. The available backbone φ and ψ dihedral angles of *l*-Tic residue in Tat1_8-9TOD and Tat1_4-5TO,8-9TOD were limited to a small range around -120° and 60°, respectively, whereas the presence of *d*-Tic enantiomer gave rise to slight increase in population of distinct local conformations. Thus, based on the values of φ and ψ dihedral angles of the *d*-Tic residue, the conformations of Tat1_4-5TO and Tat1_8-9TO were grouped into two families, either with φ and ψ fixed at *ca*. 95° and -150° or at 130° and -70°. In turn, the *d*-Tic^4^ in Tat1_4-5TO,8-9TOD adopted preferably a 120° φ angle, whereas ψ angle was changing in the range of *ca*. -120 to -40°.^.^ Nevertheless, according to the MD simulations, the Tic-Oic unit did not induce the turn structure in the place of its incorporation. Tat1_8-9TO analog adopted an extended conformation without any tendency for bent structures. In Tat1_4-5TO peptide, a single β-turn appeared exactly on the opposite terminus of the molecule than the Tic-Oic template, i.e. in the fragment Gln^7^-Arg^8^-Arg^9^-Arg^10^. This proposed β-turn was not stabilized by hydrogen bonds and was found in only *ca*. 40% of the structures (Table 2).

There were no β-turn structures detected in the peptides containing the Tic-*d*-Oic unit, i.e. Tat1_8-9TOD and Tat1_4-5TO,8-9TOD. Only a single inverse γ-turn with Arg^6^ at its apex was observed for the latter. In the case of Tat1_A4-5,8–9, a single non-hydrogen-bonded β-turn of type I or IV occupied a fragment Ala^4^-Ala^5^-Arg^6^-Gln^7^ of the peptide.

The average radii of gyration (Rg, the root mean square distance of the heavy atoms from their common center of gravity [62,63]) were 8.4, 9.7, 7.7, 8.3 and 9.5 Å, for Tat1_4-5TO, Tat1_8-9TO, Tat1_8-9TOD, Tat1_4-5TO,8-9TOD, and Tat1_A4-5,8–9, respectively. A lower value of Rg calculated for Tat1_8-9TOD was comparable with that obtained for Tat1 (7.5 Å) [36] and suggests a more compact structure than in other analogs.

The modification with neutral residues changed significantly the electrostatic potential of the peptides in comparison to Tat1 (Fig 6). Among the peptides studied, the Tat1_4-5TO,8-9TOD and Tat1_A4-5,8–9, due to replacement of as many as four positively charged residues with neutral residues, displayed the lowest total positive charge (+4). However, for the former the positively charged centers are rather concentrated on one side of the molecule, as for the other Tic-Oic modified peptides, while for the latter they seem to be distributed randomly over the whole peptide. Importantly, due to the extended conformation, all the residues were strongly water-exposed. The solvent-accessible surface area of each residue exceeded 30% with only two exceptions, i.e. Tic^8^ in Tat1_8-9TOD and Gln^7^ in Tat1_4-5TO,8-9TOD (S8 Fig).

## Discussion

Since the proteasome is an attractive drug target there have been multiple attempts to find the best way to inhibit its proteolytic activity. The preference in the search for suitable compounds at first was directed toward short peptides equipped with a reactive electrophilic group that rendered the N-terminal threonine inactive. This approach resulted in a discovery of several peptide inhibitors that have been enormously useful in further deciphering of physiological role of the proteasome. The crown achievement of this strategy was a fast track introduction of bortezomib, a dipeptide equipped with a boronate group, to fight against advanced myeloma. The role of a peptide moiety in this design was to precisely bring the war head to its designated target. Since the reactive group chemically modifies the catalytic residue of the essential enzyme, there is a strong possibility of irreversible poisoning of healthy cells and off target biomolecules. To limit such effects, a line of inhibitors has been introduced that still compete with substrates for the active centers but they achieve their goal without modification of the catalytic residues. They represent a broad range of usually short peptides build with unnatural or modified amino acids that render the compounds indigestible by the proteases [64]. Finally, the third group of inhibitors takes advantage of the allosteric regulation of the 20S core activity. Currently, two main modes of allosteric regulation are explored. The more popular way employs the binding sites of the proteasome activators located on the α face to interfere with a signaling mostly from the gate to active centers. The second group focuses on hampering interactions between the α and β rings.

Tat1 peptide is derived from the binding domain of HIV-1 Tat protein that competes with the 11S activator, therefore it likely binds to the α face of the core. However, its exact binding site(s) remain elusive. Our previous studies established that Tat1 is a non-competitive inhibitor of 20S proteasome. Although in molecular modeling it predictably shows a high level of structural flexibility, it also exhibits a marked propensity to form two β-like turns. Here, we set out to rigorously test the hypothesis that the turns are at the core of the inhibitory properties of Tat1. The sequence of Tat1 is quite monotonous with two blocks of basic residues separated by a single glutamine residue and flanked with a single glycine on its amino terminus and Pro-Ser sequence on its carboxyl terminus. Long side chains of arginine and lysine residues somewhat limit spatial flexibility of the peptide. Not surprisingly, we had to apply alanine scans involving multiple consecutive residues to disable the quite impressive inhibitory potency of Tat1 and to identify two potential pharmacophores. This way, we successfully confirmed the previously suggested with the MD studies positioning of hot spots between residues 4–6 and 8–10. Indeed, substitution of residues within these regions with alanines led to at least 5 fold increase of IC_50_ measured for ChT-L activity of SDS activated 20S proteasome. All the above differences in IC_50_, although respectable, may not seem very impressive. However, these peptides are inherently flexible and sequentially monotonous therefore their residues likely substitute each other in interactions with the proteasome. Stiffening of Tat1 structure would restrict such flexibility. If the stiffening is accompanied by the proper exposure of the basic side chains, it will allow for not only retaining but even enhancement of the inhibitory capacity. Following this lead, we introduced a set of peptidomimetic residues Tic and Oic in the identified hot spots. The role of the Tic-Oic moiety was to induce or stabilize β-type turns strongly suggested by the MD. Unexpectedly, simultaneous incorporation of the beta turn inducer in the two hot spots did not bring substantial improvement to IC_50_. Moreover, the TO moiety at the position 4–5 helped with only a modest gain in IC_50_. We observed a significant decrease of IC_50_ only when the TO group was placed in the position 8–9. Importantly, this site was sensitive to stereochemistry of the moiety since incorporation of the TOD improved IC_50_ about 30% when compared with the TO group. Based on these results we propose that the positioning of the stable β-turn at the C-terminus is beneficial for inhibitory potency of the peptide. Although suggested by the MD simulations of Tat1, the simultaneous presence of stabilized rigid turns in both pharmacophore positions is not favorable. This indicates the improved design of Tat1 derivatives that includes a flexible longer N-terminal arm with a single β-turn placed close to the C-terminus capped with the Pro-Ser dipeptide. We predict that for correct binding either this arm should stay flexible or its correct structure is induced only when the β-turn docks.

The 12-mer of Tat1 is built with 8 basic residues grouped in sets of 5 and 3 members. Therefore, we expect that such accumulation of the positive charge has to play an important role in both maintaining preferred structure of the peptide and in its interactions with the target protein. Indeed, replacement of the charged residues with a much shorter and hydrophobic alanine partially removes steric hindrance of a long aliphatic chain decorated with a bulky guanidine group. Apparently, a high mobility decreases chances for the peptide to productively fit into the binding pocket(s). Additionally, the peptide may lose its specificity and probe alternative binding sites that have no or even an opposite effect on the peptidase activity. We are currently working toward solving a molecular structure of the proteasome with the bound Tat1 peptide to determine location of the binding pocket/s specific for allosteric inhibitors.

Intriguingly, we first identified the importance of structural dynamics of this peptide, when we compared the FTIR spectra collected in liquid and films. Contrary to our predictions, the Tat1 derivatives immobilized in a film or dissolved in D_2_O showed a clear propensity to form β-turns. The differences between the structures of Tat1 were surprising since we did not observe such dependence in a Tat2 structure. This linear peptide of identical length as Tat1 is also a non-competitive inhibitor of 20S proteasome, albeit with twice as high IC_50_ [37]. However, Tat2 contains an acidic Asp residue in its C-terminal part, which may influence the structural stability by formation of a salt bridge with one of several basic Arg/Lys residues present in the N-terminal part of the peptide. Since in the case of Tat1 there are no residues that could stabilize the structure through charge pairing, the peptide is in a random coil conformation when dissolved in water. On the other hand, however, Tat1 and its analogs seem to exist at the boundary of a more stable structure since limiting their flexibility by solvent evaporation or tightening the bonds due to the H/D exchange, enabled the peptides to achieve more ordered architecture with evident preferences for a turn conformation.

We recorded also CD spectra that reflect the averaged conformations of the peptides. The spectra were surprisingly sensitive to the position and stereochemistry of the introduced Tic-Oic substitution. We noticed that contrary to the CD spectrum of Tat1_8-9TO, representing random coil structure (Fig 4), Tat1_8-9TOD peptide preferentially adopted the βII turn conformation. This observation suggests that the change of stereochemistry around the Tic-Oic moiety in position 8–9 profoundly influenced flexibility of the peptide making it more rigid. Unexpectedly, the *d*-Tic,*l*-Oic unit, which stabilized an extended PPII conformation while present alone in the region 4–5, in the presence of 8-9TOD altered the bias from the βII toward the βI turn [44]. We concluded that it is necessary to take into account the complete structural context including both pharmacophore regions, to define the conformational preferences of Tat1-based modulators.

In our attempt to better structurally characterize the peptides we showed using NMR spectroscopy that the *trans* geometry of a peptide bond was preferred around the proline-like Tic and Oic residues. Since no measurable ROE cross-peaks were observed between the successive backbone amide groups (S6 Fig), we could not ascribe folded conformation to the major conformers of any TO/TOD peptides. Based on the strong d_αN_(*i*,*i+1*) and d_αδ_(*i*,*i+1*) ROE cross-peaks and the values of ^3^J_HNHα_ coupling constant we suggested an extended, the most probably PPII conformation, for the major conformers of Tat1_4-5TO, Tat1_8-9TO, Tat1_8-9TOD and Tat1_4-5TO,8-9TOD.

We performed the MD simulations using NMR-derived time-averaged distance and dihedral angle restraints. In general, they confirmed a high propensity of the studied compounds to adopt an extended (β-strand and/or PPII) conformation. MD proposed also turn structures, such as a β-turn involving the fragment Gln^7^-Arg^8^-Arg^9^-Arg^10^ in Tat1_4-5TO; an inverse γ-turn with Arg^6^ at its apex in Tat1_4-5TO,8-9TOD; and a β-turn of type I or IV occupying a fragment Ala^4^-Ala^5^-Arg^6^-Gln^7^ in Tat1_A4-5,8–9, but none of them was hydrogen bonded. Flexibility of the peptides due to the lack of structure stabilizing intramolecular hydrogen interactions, as well as lack of clear relationship between the structural preferences and affinity toward the proteasome seems to advocate for the induced-fit binding of the TO/TOD Tat1 analogs to the 20S proteasome. On the other hand, while NMR and FTIR spectroscopy did not reveal any turns for Tat1_8-9TOD nor Tat1_4,5TO,8-9TOD (Table 2, S9 Fig), CD spectra of these two peptidomimetics displayed distinct spectral features of the βII and βI turn, respectively (Fig 3). One of possible explanations for this discrepancy is concentration-induced conformational changes. NMR and FTIR experiments required concentration of the sample about 25 to 100 times higher than CD spectroscopy. Likely the concentration of the analyte for the very polar and basic Tat1 analogs is crucial to stabilize the extended structure with the extensive solvent-mediated interactions. Significantly lower peptide concentrations used in the CD experiments were closer to the concentrations of the modulators employed in the biological tests what implies that the CD derived structural data may be more relevant when establishing the structure-activity correlation for the case of Tat1 analogs.

Interestingly, Tat1 peptide is not the sole example of a basic, Arg-rich proteasome modulator. The most notable examples are Pro and Arg-rich peptides (PR peptides), which inhibit the activated 20S proteasome *in vitro* at high nanomolar/low-micromolar concentrations, and preferentially inhibit degradation of selected protein substrates *in vivo*. The best studied and shortest 11-residue PR11 may contain a structural turn and is hypothesized to bind at the outer edge of the proteasomal α ring [24]. Another example is octa-arginine, inhibiting the proteasome peptidase activity *in vitro* with high-nanomolar IC_50_, and leading to accumulation of proteasome substrates in cultured cells [65]. The binding site(s) and mode(s) of this mixed-action inhibitor are unknown.

It remains to be established whether the introduction of β-turns *via* the Tic-Oic moiety has a unique influence on the inhibitory properties of Tat1 peptide or it could be recapitulated with other turn inducers. Our preliminary exploration of the chemical space indicates that other turn inducers can to some extent substitute for Tic-Oic [66]. The structure of the region 4–6 is important for inhibition of the 20S proteasome. However, it is not clear if the region would remain flexible after docking to the proteasome or if it strikes a unique pose. Apparently, stiffening of the 4–6 region before the peptide docking is not deleterious for the inhibitory potency but it does not improve it either. Finally, although we are confident that our peptides are not digested by proteasome at a MS-detectable rate, they may still influence other than ChT-L active centers and the digest of protein substrates in a hard to predict way. We currently work fervently toward tackling these questions.

## Conclusions

In this work we attempted to identify and improve pharmacophores of Tat1 peptide which constitutes an important example of a non-competitive, likely allosteric inhibitor of the 20S proteasome. We found a single β-turn promoting region specifically positioned close to the C-terminus in the sequence of Tat1, which when stiffened with the β-turn inducing Tic-Oic moiety produced a substantially improved inhibitor. The accumulated spectroscopic and biological data for Ala and Tic-Oic substituted analogs of Tat1 suggests a complex interplay of the structural flexibility and charge in regulation of the peptides inhibitory potency. The current data also support the possibility that due to the structural flexibility of these peptides they can follow an induced fit pathway when they probe the 20S proteasome as a potential receptor. It appears that the Tat1 constitutes a unique structural scaffold on which we plan to build further allosteric inhibitors of 20S proteasome that could be expanded into specific regulators of this essential protease.

## Materials and Methods

### Synthesis of Ta1 analogs

The alanine analogs of Tat1 peptide were synthesized on a pre-loaded Wang resin (100–200 mesh, loading capacity 0.6 mmol/g) purchased from Advanced ChemTech (USA). The synthesis was performed on an automated peptide synthesizer 443A (Applied Biosystems, USA) utilizing a standard Fmoc/tBu strategy. Peptides Tat1_4-5TO, Tat1_8-9TO, Tat1_8-9TOD and Tat1_4-5TO,8-9TOD were obtained using a microwave-assisted manual Fmoc/tBu methodology in a microwave reactor Plazmatronika RM800 (Ertec Inc., Poland) equipped with a reaction vessel with nitrogen agitation.

Crude peptides were purified by reversed-phase high-performance liquid using a C8 semi-preparative Luna column (21.2 x 250 mm, 5 μm; Phenomenex). A linear gradient of acetonitrile in 0.1% aqueous trifluoroacetic acid or acetonitrile in 0.1 M triethylamine phosphate buffer, pH 3.0, was used as a mobile phase. The molecular weights of the peptides were ascertained by matrix-assisted laser desorption/ionization time-of-flight mass spectrometry or electrospray ionization ion trap time-of-flight liquid chromatography mass spectrometry with C12 Jupiter Proteo column (150 x 2 mm, 4μ, 90Å; Phenomenex). The purity of the synthesized compounds was confirmed by analytical reversed phase chromatography using a C8 Kromasil column (4.6 x 250 mm, 5 μm) and a 30 min linear gradient of 5–80% acetonitrile in 0.1% aqueous trifluoroacetic acid. UV absorption was observed at λ = 223 nm.

### Activity assays

Influence of the designed peptides on catalytic properties of the 20S proteasome was tested using the proteasome at a final concentration of 1.5 nM, isolated from human erythrocytes (Enzo Life Sciences) and activated with 0.01% sodium dodecyl sulfate. Succinyl-Leu-Leu-Val-Tyr-4-methylcoumarin-7-amide (SucLLVY-MCA) was employed as a model fluorogenic substrate to determine the activity of ChT-L peptidase. All assays were performed in the 96-well plate format using a reaction volume of 100 uL. Tests were carried out in 50 mM Tris/HCl, pH 8.0 containing 0.5 mM ethylenediamine tetraacetic acid. Stock solutions of the substrates and the tested peptides were prepared by dissolution of the lyophilized compounds in dimethyl sulfoxide (DMSO). The content of DMSO never exceeded 3% of the final reaction volume. Compounds were tested in the concentration range of 0.05–10 μM. The substrate was added at 100 μM final concentration (1% of the volume). The release of an aminomethyl coumarin (AMC) group was followed by measuring fluorescence emission at 460 nm in 2 min. intervals for up to 60 min. at 37°C (Fluoroskan Ascent). The peptidolytic activity was calculated as nanomoles of the released AMC product per mg of CP per second. The half maximal inhibitory concentration expressed in μM concentration was calculated with SigmaPlot 12.3 where exponential equation estimates based on fitting of two or three logistic parameters were employed.

### Fourier Transform Infrared Spectroscopy

The peptides were dissolved in H_2_O to a final concentration of 20 mg/ml. Measurements were performed in a demountable cell equipped with CaF_2_ windows and 6 μm spacer. For the film mode 15 μl of the peptide solution was spotted on a CaF_2_ window and the solvent evaporated under a stream of gaseous nitrogen. FTIR spectra were collected with a Bruker IFS66 spectrophotometer (Bruker Inc., Germany) using a deuterated triglycine sulfate detector. During data collection the spectrometer was continuously purged with dry air free of CO_2_. Typically, 240 scans at 4 cm^-1^ resolution were averaged for each spectrum. At least three independent measurements were performed for each sample. Data processing was executed with GRAMS 5.0 (Galactic Enterprises). The spectra of water and pure TFA were subtracted from the peptides spectra, as it was described [37]. The spectral 2^nd^ derivative was calculated with the application of a 9-point cubic Sawitzky-Golay smoothing function.

### Circular dichroism spectroscopy

For CD measurements, 1 mm path length quartz cuvettes were used. Protein samples were dissolved in H_2_O or in 50 mM Tris/HCl, pH 8.0 at a concentration 0.2 mg/ml. Spectra were acquired at 25°C on a Jasco J-815 spectrometer in a range 185–260 nm for aqueous solutions and 195–260 nm for solutions of the peptides in the buffer. Each spectrum is a result of three independent measurements.

### Nuclear Magnetic Resonance

The NMR samples were dissolved in H_2_O:D_2_O (9:1, v/v) (Tat1_A4-5,8–9) or partially deuterated phosphate buffer at pH 7.4 (Tat1_4-5TO, Tat1_8-9TO, Tat1_8-9TOD and Tat1_4-5TO,8-9TOD). Due to low quality of NMR spectra at pH 7.4 for the two latter, the pH was decreased to 4.5 by addition of the diluted acetic acid. The concentration of the samples was 5 mM in the case of peptides modified with Tic-Oic moiety and 10 mM in the case of Tat1_A4-5,8–9. For all peptides with Tic-Oic unit, NMR data sets were acquired at 301 K using a Varian Unity Plus 500 MHz operated at 11.7 T (^1^H resonance frequency 499.83 MHz). With Tat1_A4-5,8–9, due to a poor amide proton resonance dispersion, the 2D NMR spectra were recorded at 283 K and 298 K on a Bruker AVANCE III 700 MHz spectrometer (16.4 T, ^1^H resonance frequency 700.62 MHz). Proton resonance assignments were achieved by analysis of the 2D total chemical shift correlation spectroscopy (TOCSY) [67], the double-quantum filtered correlation spectroscopy (DQF-COSY) [68], the nuclear Overhauser effect spectroscopy (NOESY) [69], the rotating-frame Overhauser enhancement spectroscopy (ROESY) [70,71] as well as the gradient heteronuclear single quantum coherence experiment (^1^H-^13^C gHSQC) [72–74]. The volumes of cross peaks were picked up for the ROESY spectra with a mixing time of 300 ms. In the case of Tat1_A4-5,8–9, the ROESY spectrum recorded at 283 K was considered due to better signal dispersion. Vicinal coupling constants, ^3^J_HNHa_, were assigned using DQF-COSY. For Tat1_A4-5,8–9, the coupling constants were also measured from 1D NMR spectrum and the final values were obtained by averaging values from both DQF-COSY and 1D NMR spectra.

All chemical shifts were referenced with respect to external sodium 2,2-dimethyl-2-silapentane-5-sulfonate using a Ξ = 0.251449530 ratio for indirectly referenced ^13^C resonances [75]. The data were processed using VNMR (Varian Inc., Palo Alto, CA) and NMRPipe [76] and analyzed with the SPARKY software [77].

### Molecular Dynamics Simulations

Molecular dynamics simulations were carried out with the parm99 force field in the AMBER11.0 package [78]. The valence geometry of the residues not specified in the standard AMBER database was parameterized as recommended by the AMBER 11.0 manual. Specifically, these residues were modeled using bond lengths, the valence and torsion angles of appropriate residues and compatible molecular segments taken from the CSDS [79] database. The point charges were optimized by fitting them to the *ab initio* molecular electrostatic potential (6-31G* basis set, GAMESS’04 [80]—*ab initio* molecular electronic structure program) for three different conformations of every nonstandard residue, followed by consecutive averaging the charges over all conformations, as recommended by the RESP protocol [81].

The structures of the major species of the peptides were computed using molecular dynamics simulations with explicit water and NMR-derived restraints. All MD simulations were run with the SHAKE algorithm (with a tolerance of 0.0005 Å) to constraint covalent bonds involving hydrogens with a 2-fs time step using periodic boundary conditions, and a temperature of 303 K (Berendsen temperature regulation). The electrostatic interactions were evaluated using the PME summation method with cutoff of 9 Å. All models for the MD simulations were constructed using LEAP (AMBER 11 package). The initially random structure of a peptide was embedded into a TIP3P water box enlarged by 10 Å in each direction from the solute. Each system was neutralized by a minimum amount of chloride ions. To equilibrate the solution density, the initial simulations were carried out in NTP ensemble at 1 atm (1 ns). The final density was close to 1.0 g/mL. In the next step, the NTV MD simulations (4 ns) with time-averaged distance restraints and dihedral angle restraints derived from the NMR spectroscopy were employed. The interproton distances were restrained with the force constants f = 50 kcal/(mol x Å^2^), and the dihedral angles with f = 2 kcal/(mol x rad^2^). The geometry of the peptide groups was kept fixed according to the NMR data (f = 50 kcal/(mol x rad^2^)). The inter-proton distances were calculated on the basis of ROEs intensities by the CALIBA algorithm of the DYANA [82] program. The upper limit distance restraints and the ^3^J_HNHα_ vicinal coupling constants were used in the HABAS algorithm of the DYANA package to generate φ, ψ and χ ^1^ dihedral angle restraints and stereospecific assignments.

The coordinates were collected every 2000^th^ step. The results were analyzed using Ptraj program from the AMBER 11 package. The conformations obtained in the last 800 ps of simulations were considered in further analysis. The final structures were visualized by MOLMOL [83].

### Microscale thermophoresis

The human 20S proteasome was labeled using monolith NT^TM^ Protein Labeling Kit RED-NHS (NanoTemper Technologies), according to the protocol provided by the supplier. During the measurements concentration of the labeled protein was kept constant at 3 nM. The peptides used as unlabeled binding partners were titrated in 3:1 dilutions in the concentration range of 0.2 to 120 μM (dependently on K_d_ values). Concentrations below 0.2 μM were tested as well but thermophoretic effects were not observed. The binding reactions were carried out in 50 mM Tris/HCl buffer containing 0.5 mM EDTA, pH 8.0 with an addition of sodium dodecyl sulfate at the final concentration of 0.005%. Measurements were performed in standard treated capillaries on a Monolith NT.115 system (NanoTemper Technologies). Capillaries were illuminated at 25°C with 80% LED power for 5 s. Thermophoresis was tracked for 30 s in three laser intensity sets: 80, 60 and 40%, back-diffusion was observed for 5 s. Fluorescence intensities were measured at 6–8 s and 34–36 s, and fluorescence intensity ratios of hot/cold were used as the assay readout. K_d_ values were determined using the internal evaluation software.

### Peptide degradation tests

Susceptibility of the designed modulators to degradation by the 20S proteasome was examined by reversed-phase high-performance liquid chromatography (S10 Fig). The compounds were dissolved in water or DMSO to 10 mM concentration and then diluted with 25 mM Tris buffer containing 0.25 mM EDTA (pH 8.0) to the final concentration of 1 mM. 2.5 μg of human 20S proteasome (Enzo Life Sciences) was added to the mixture, which then, after adjustment of its final volume to 100 μl, was incubated at 37°C for 3 h. The reaction was stopped by adding 5 μl of 10% aqueous trifluoroacetic acid. Next, 40 μl of the mixture, along with the control not containing the 20S, were injected onto C8 Kromasil column (4,6 x 250 mm, 100 Å, 5 μm). The linear gradient of acetonitrile in 0.1% aqueous trifluoroacetic acid was performed with SIL-20AHT UFLC system equipped with SPD-M20A PDA detector (SHIMADZU), and the elution was observed at the wavelength of 223 nm.

## Supporting Information

S1 Data Inhibitory data of Tat1 analogs against human 20S proteasome. (XLSX)

S2 Data Thermophoresis data. (XLSX)

S1 Fig Relative chymotrypsin-like activity of human 20S proteasome in the presence of Tat1 inhibitor or its Ala-scan analogs. The peptides concentration was either 0.1 or 1 μM.
(PDF)

S2 Fig Comparison of IC_50_ and K_d_ values for the selected peptides with modifications of the postulated pharmacophore regions by either Ala or TicOic substitutions. (PDF)

S3 Fig Second derivative of the FTIR spectra of the single Ala-scan peptides in H_2_O (A) and in a hydrated film mode (B). (PDF)

S4 Fig Second derivative of the FTIR spectra of Tat1A4-6 and Tat1A8-10 Ala-scan peptides in H_2_O and D_2_O. (PDF)

S5 Fig Normalized CD spectra in water for the selected peptides with single and multiple Ala substitutions. (PDF)

S6 Fig Inter-residue ROE interactions for Tat1_4-5TO (A), Tat1_8-9TO (B), Tat1_8-9TOD (C), Tat1_4-5TO,8-9TOD (D) and Tat1_A4-5,8–9 (E). (PDF)

S7 Fig Scatter plots of the dihedral angles (ϕ and ψ of all residues and all conformations of Tat1_4-5TO (A), Tat1_8-9TO (B), Tat1_8-9TOD (C), Tat1_4-5TO,8-9TOD (D) and Tat1_A4-5,8–9 (E) calculated by MD simulations with time-averaged restraints. (PDF)

S8 Fig The average surface accessibility of amino acid residues in the peptides studied calculated with MOLMOL using a solvent probe radius of 1.4 Å. The conformations obtained in the last 800 ps of MD simulations with time-averaged distance restraints and dihedral angle restraints.
(PDF)

S9 Fig Second derivative of Tat1_8-9TOD and Tat1_4-5TO,8-9TOD FTIR spectra recorded in water. (PDF)

S10 Fig Susceptibility of Tat1 and its analogs with the best inhibitory capacity to 20S proteasome degradation. (PDF)

S1 Table The synthesized analogs of Tat1 with multiple amino acid residues exchanged into Ala or Tic-Oic moiety. (PDF)

S2 Table Dissociation constants K_d_ calculated on the basis of the thermophoresis curves. (PDF)

S3 Table Proton chemical shifts and the vicinal coupling constants of Tat1_A4-5,8–9 in H_2_O/D_2_O (9:1) at 298 K. (PDF)

S4 Table Proton chemical shifts and the vicinal coupling constants of Tat1_4-5TO in phosphate buffer pH 7.4 at 301 K. (PDF)

S5 Table Proton chemical shifts and the vicinal coupling constants of Tat1_8-9TO in phosphate buffer pH 7.4 at 301 K. (PDF)

S6 Table Proton chemical shifts and the vicinal coupling constants of Tat1_8-9TOD in phosphate buffer with the reduced pH (pH 4.5) at 301 K. (PDF)

S7 Table Proton chemical shifts and the vicinal coupling constants of Tat1_4-5TO,8-9TOD in phosphate buffer with the reduced pH (pH 4.5) at 301 K. (PDF)
