# Supplementary material for: Interplay between Structure and Charge as a Key to Allosteric Modulation of Human 20S Proteasome by the Basic Fragment of HIV-1 Tat Protein
Source: PLoS One. 2015 Nov 17;10(11):e0143038. doi: 10.1371/journal.pone.0143038 (PMC4648528; doi:10.1371/journal.pone.0143038)
Supplement: S1 Fig — The peptides concentration was either 0.1 or 1 μM. (PDF) [file pone.0143038.s003.pdf]

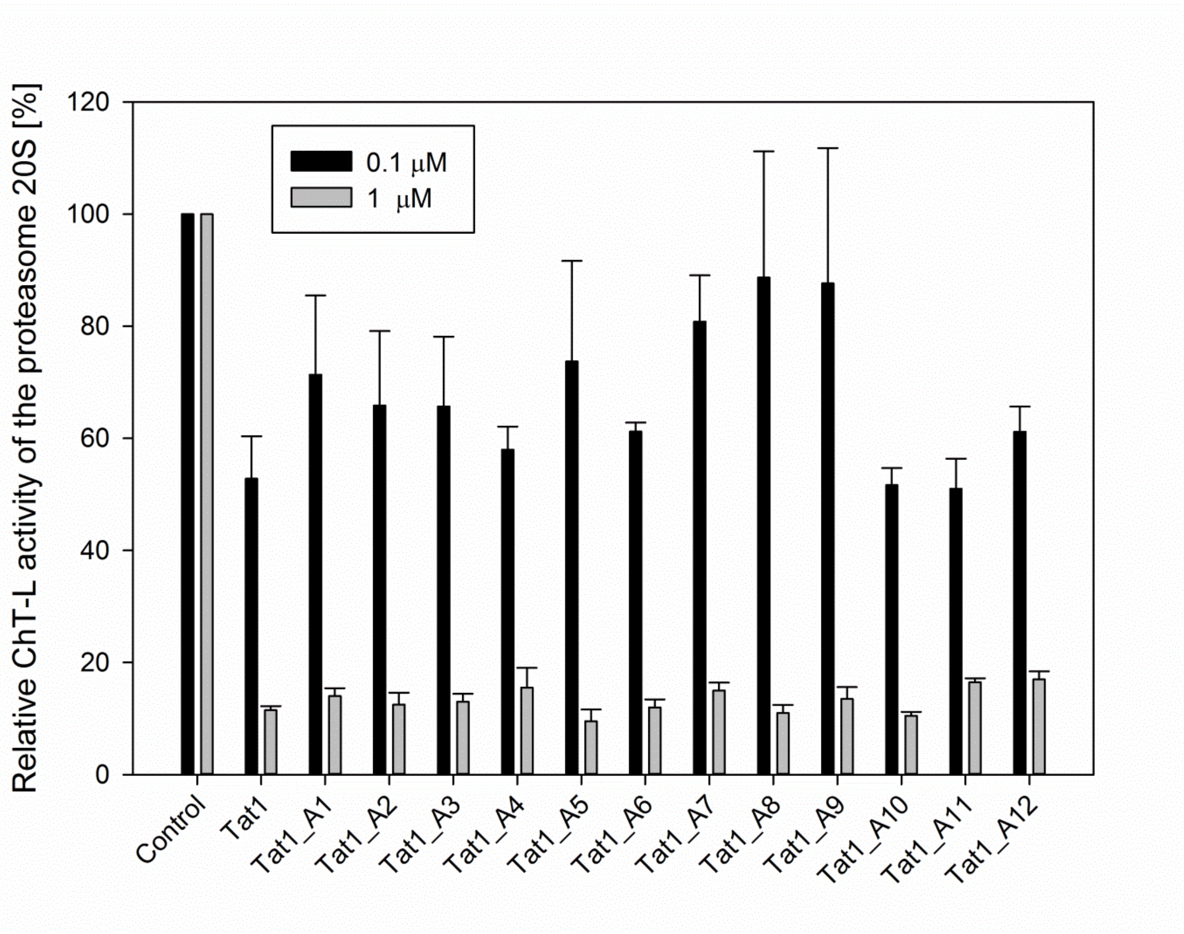

S1 Fig. Relative chymotrypsin-like activity of human 20S proteasome in the presence of Tat1 inhibitor or its Ala-scan analogs. The peptides concentration was either 0.1 or 1 μM.
