# Supplementary material for: Interplay between Structure and Charge as a Key to Allosteric Modulation of Human 20S Proteasome by the Basic Fragment of HIV-1 Tat Protein
Source: PLoS One. 2015 Nov 17;10(11):e0143038. doi: 10.1371/journal.pone.0143038 (PMC4648528; doi:10.1371/journal.pone.0143038)
Supplement: S2 Fig — (PDF) [file pone.0143038.s004.pdf]

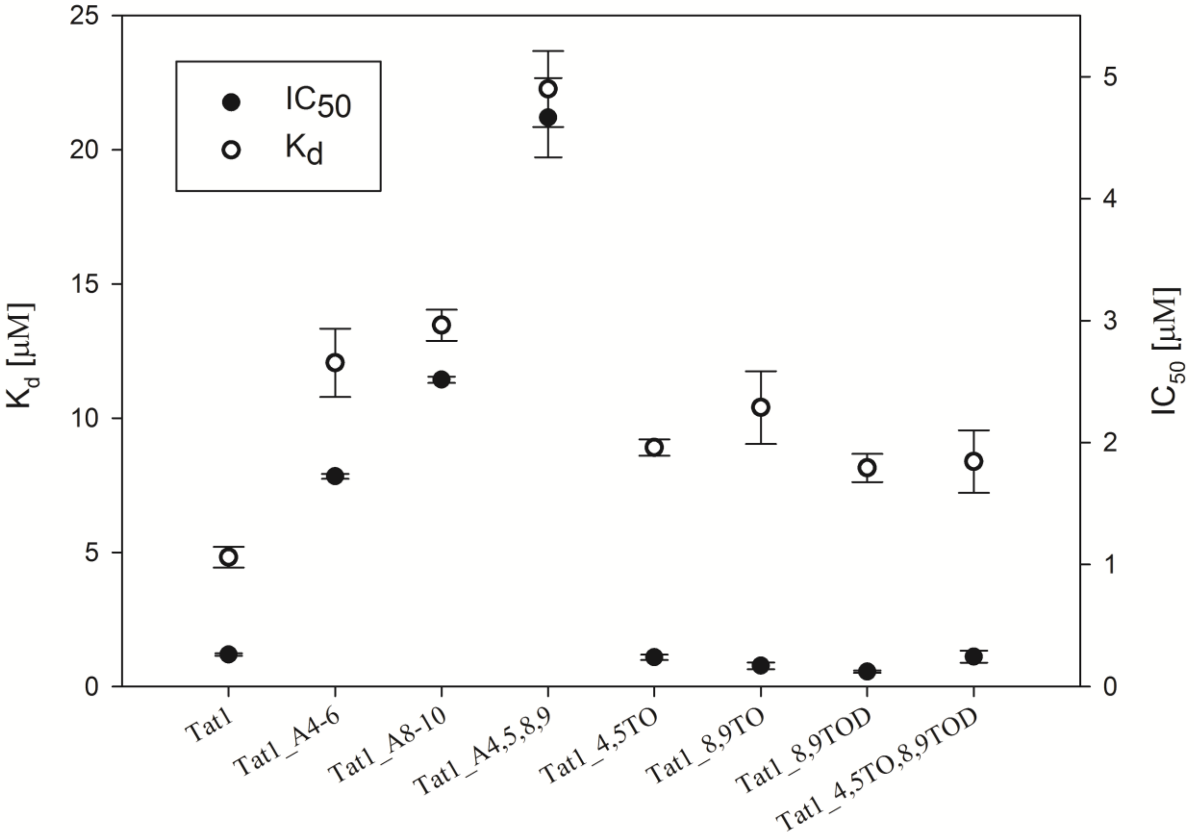

S2 Fig. Comparison of IC_{50} and K_d values for the selected peptides with modifications of the postulated pharmacophore regions by either Ala or TicOic substitutions**.**
