# Supplementary material for: Interplay between Structure and Charge as a Key to Allosteric Modulation of Human 20S Proteasome by the Basic Fragment of HIV-1 Tat Protein
Source: PLoS One. 2015 Nov 17;10(11):e0143038. doi: 10.1371/journal.pone.0143038 (PMC4648528; doi:10.1371/journal.pone.0143038)
Supplement: S3 Fig — (PDF) [file pone.0143038.s005.pdf]

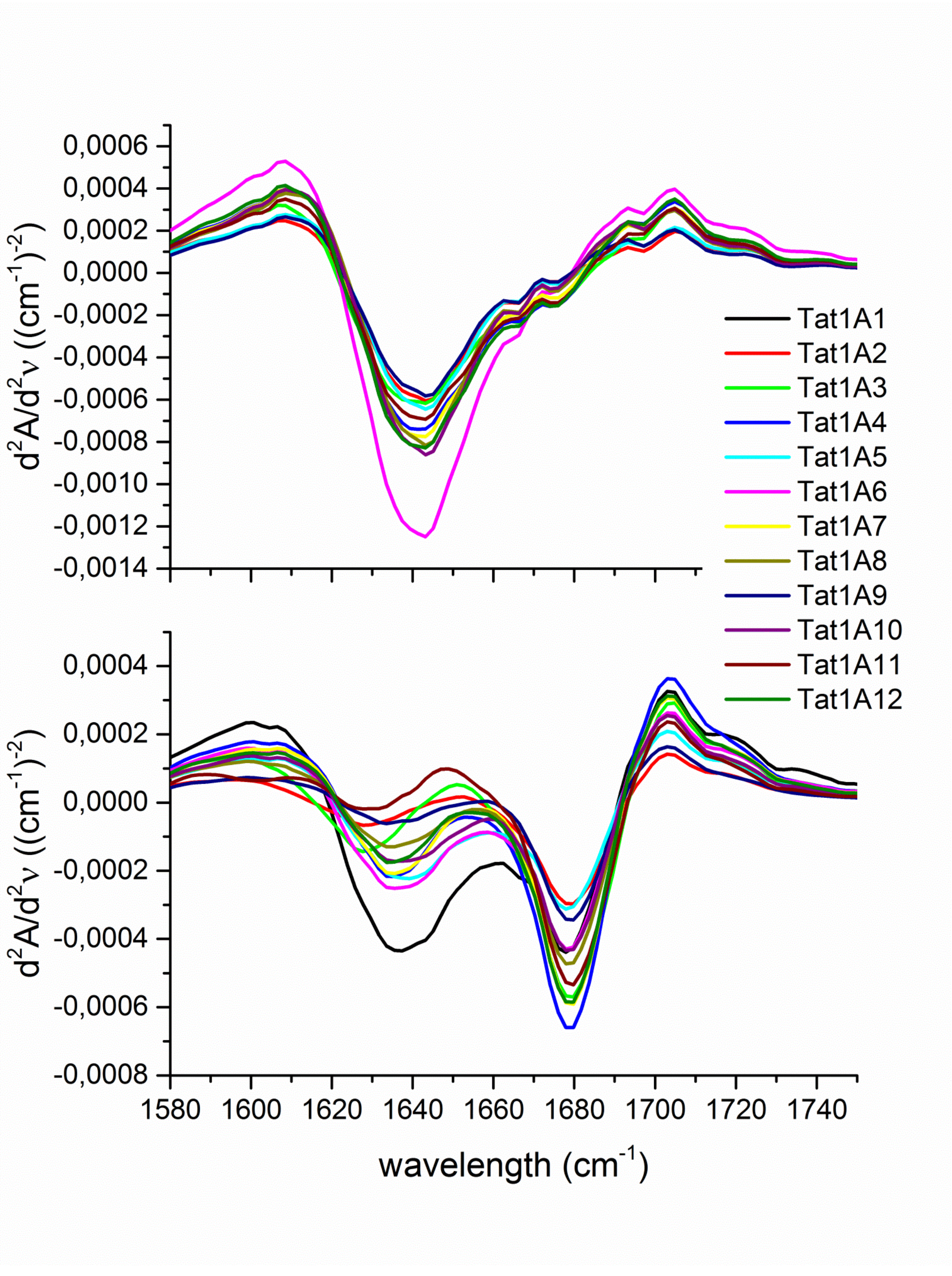

S3 Fig. Second derivative of the FTIR spectra of the single Ala-scan peptides in H_2O (A) and in a hydrated film mode (B).
