# Supplementary material for: Interplay between Structure and Charge as a Key to Allosteric Modulation of Human 20S Proteasome by the Basic Fragment of HIV-1 Tat Protein
Source: PLoS One. 2015 Nov 17;10(11):e0143038. doi: 10.1371/journal.pone.0143038 (PMC4648528; doi:10.1371/journal.pone.0143038)
Supplement: S4 Fig — (PDF) [file pone.0143038.s006.pdf]

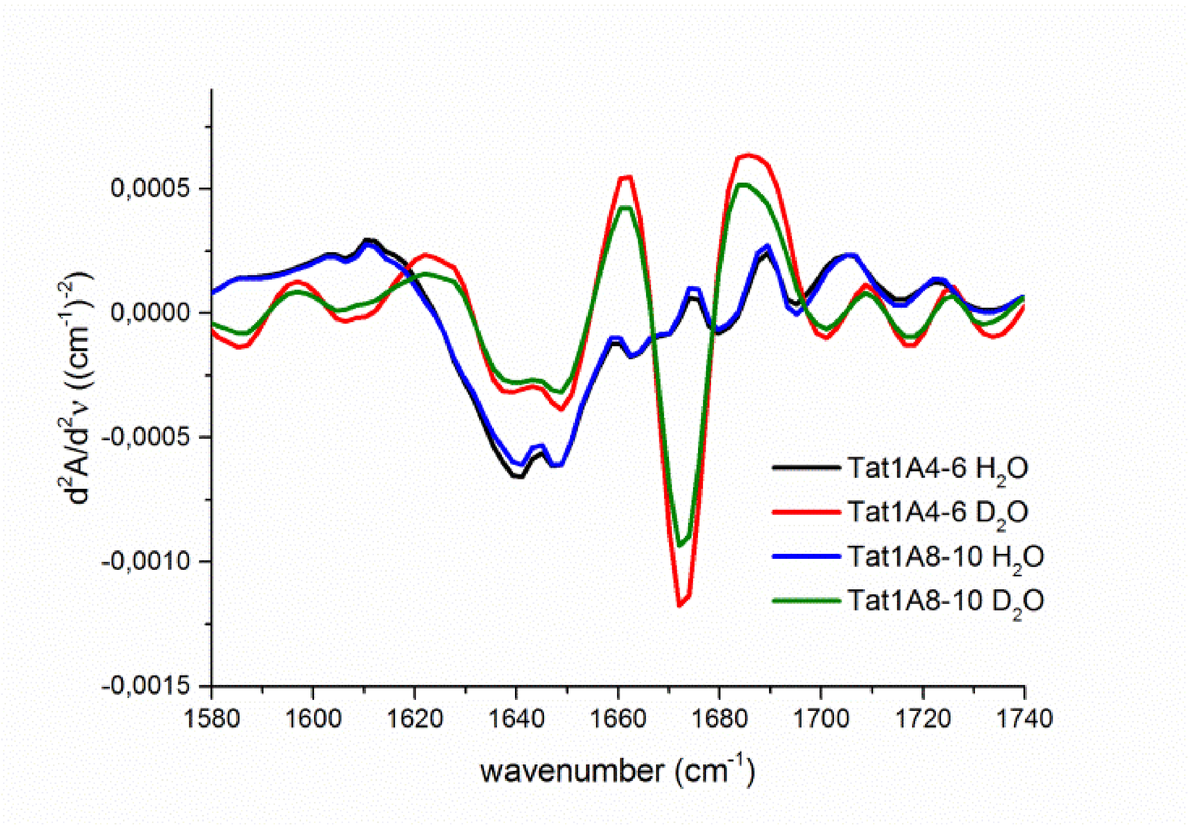

S4 Fig. Second derivative of the FTIR spectra of Tat1A4-6 and Tat1A8-10 Ala-scan peptides in H_2O and D_2O.
