# Supplementary figures and images for: Interplay between Structure and Charge as a Key to Allosteric Modulation of Human 20S Proteasome by the Basic Fragment of HIV-1 Tat Protein
Source: PLoS One. 2015 Nov 17;10(11):e0143038. doi: 10.1371/journal.pone.0143038 (PMC4648528; doi:10.1371/journal.pone.0143038)

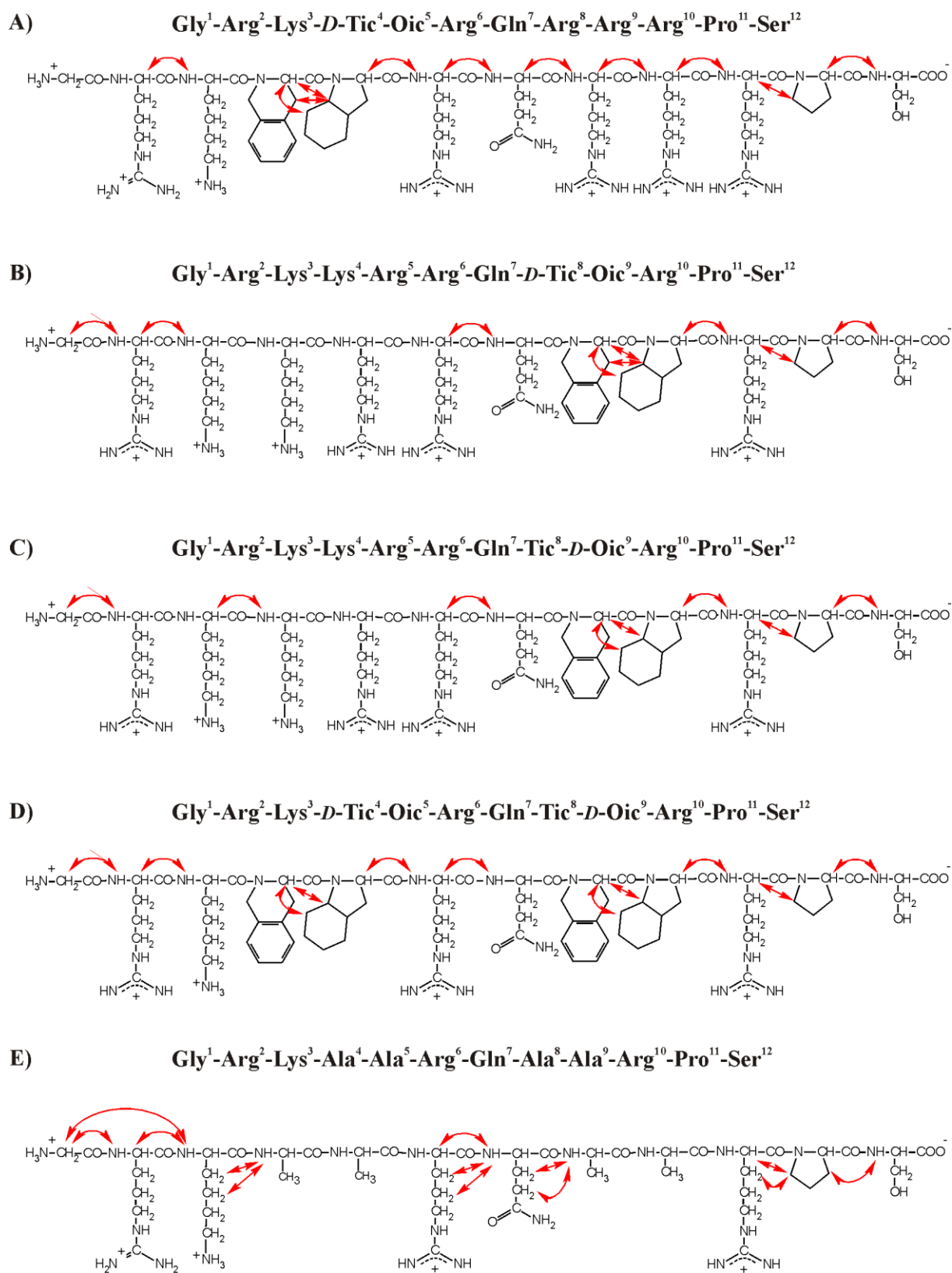

**S6 Fig.** Inter-residue ROE interactions for Tat1\_4-5TO (A), Tat1\_8-9TO (B), Tat1\_8-9TOD (C), Tat1\_4-5TO,8-9TOD (D) and Tat1\_A4,5,8-9 (E).

Supplement: S6 Fig — (PDF) [file pone.0143038.s008.pdf]

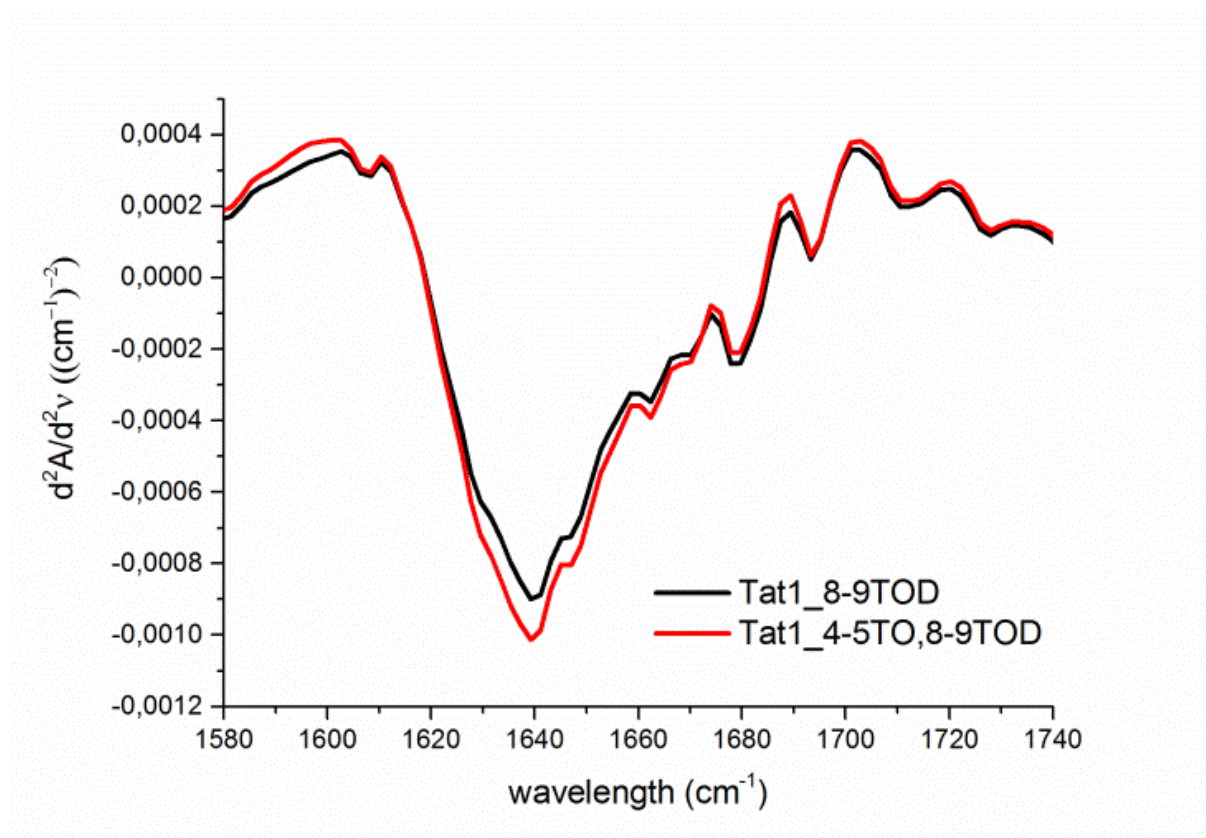

**S9 Fig.** Second derivative of Tat1\_8-9TOD and Tat1\_4-5TO,8-9TOD FTIR spectra recorded in water.

Supplement: S9 Fig — (PDF) [file pone.0143038.s011.pdf]
