# Supplementary material for: Interplay between Structure and Charge as a Key to Allosteric Modulation of Human 20S Proteasome by the Basic Fragment of HIV-1 Tat Protein
Source: PLoS One. 2015 Nov 17;10(11):e0143038. doi: 10.1371/journal.pone.0143038 (PMC4648528; doi:10.1371/journal.pone.0143038)
Supplement: S7 Fig — (PDF) [file pone.0143038.s009.pdf]

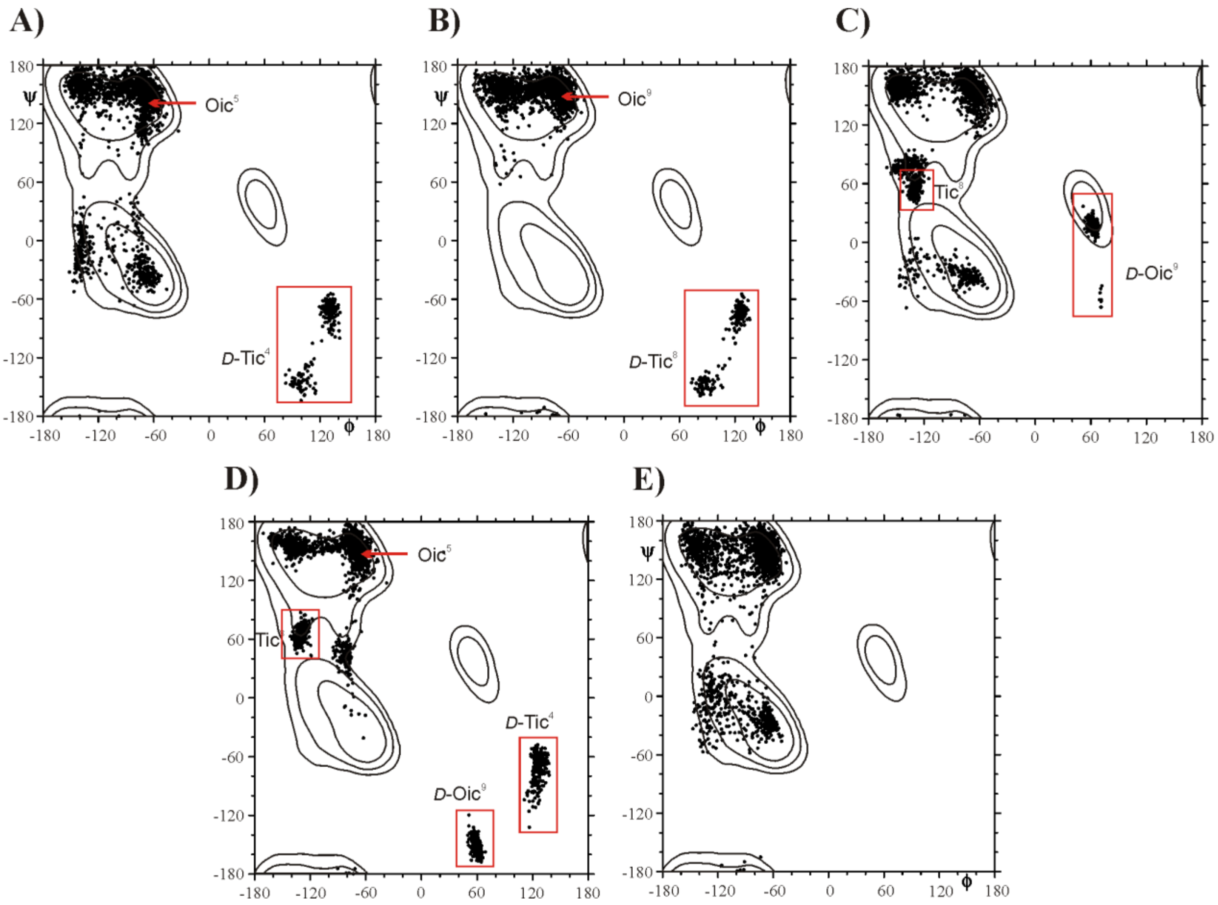

S7 Fig. Scatter plots of the dihedral angles (ϕ and ψ of all residues and all conformations of Tat1_4-5TO (**A**), Tat1_8-9TO (**B**), Tat1_8-9TOD (**C**), Tat1_4-5TO,8-9TOD (**D**) and Tat1_A4-5,8-9 (**E**) calculated by MD simulations with time-averaged restraints.
