# Supplementary material for: Interplay between Structure and Charge as a Key to Allosteric Modulation of Human 20S Proteasome by the Basic Fragment of HIV-1 Tat Protein
Source: PLoS One. 2015 Nov 17;10(11):e0143038. doi: 10.1371/journal.pone.0143038 (PMC4648528; doi:10.1371/journal.pone.0143038)
Supplement: S8 Fig — The conformations obtained in the last 800 ps of MD simulations with time-averaged distance restraints and dihedral angle restraints. (PDF) [file pone.0143038.s010.pdf]

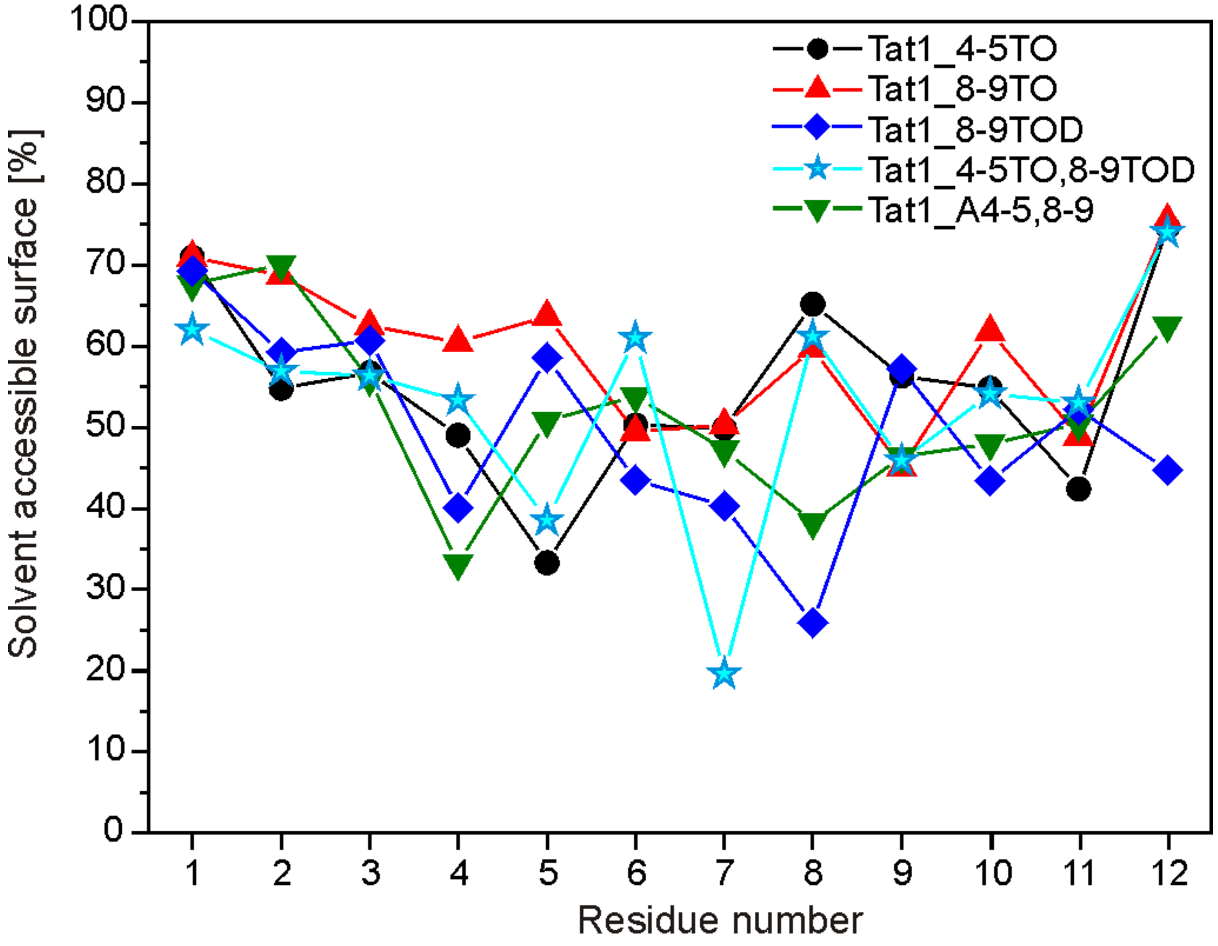

S8 Fig. The average surface accessibility of amino acid residues in the peptides studied calculated with MOLMOL using a solvent probe radius of 1.4 Å. The conformations obtained in the last 800 ps of MD simulations with time-averaged distance restraints and dihedral angle restraints.
