# Supplementary material for: Interplay between Structure and Charge as a Key to Allosteric Modulation of Human 20S Proteasome by the Basic Fragment of HIV-1 Tat Protein
Source: PLoS One. 2015 Nov 17;10(11):e0143038. doi: 10.1371/journal.pone.0143038 (PMC4648528; doi:10.1371/journal.pone.0143038)
Supplement: S10 Fig — (PDF) [file pone.0143038.s012.pdf]

Below we present the data on proteolytic stability of Tat1 and its analogs with the best inhibitory capacity. No significant degradation has been detected for any of them for up to 3 hours of incubation with the proteasome under standard conditions used in our tests. For comparison we also included the degradation profile of Tat1_A4-5,8-9, which was not able to inhibit the proteasome activity. We found this peptide to be partially degraded by the proteasome.

Tat1:

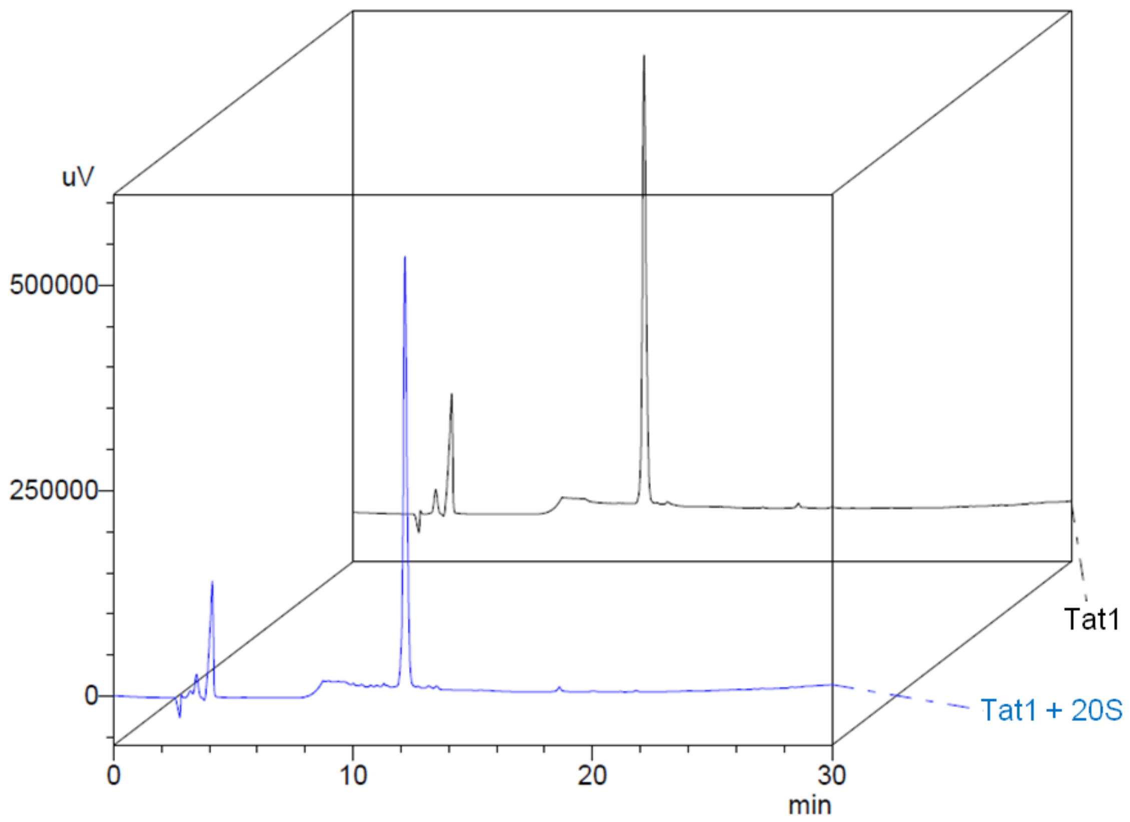

Tat1_4-5TO:

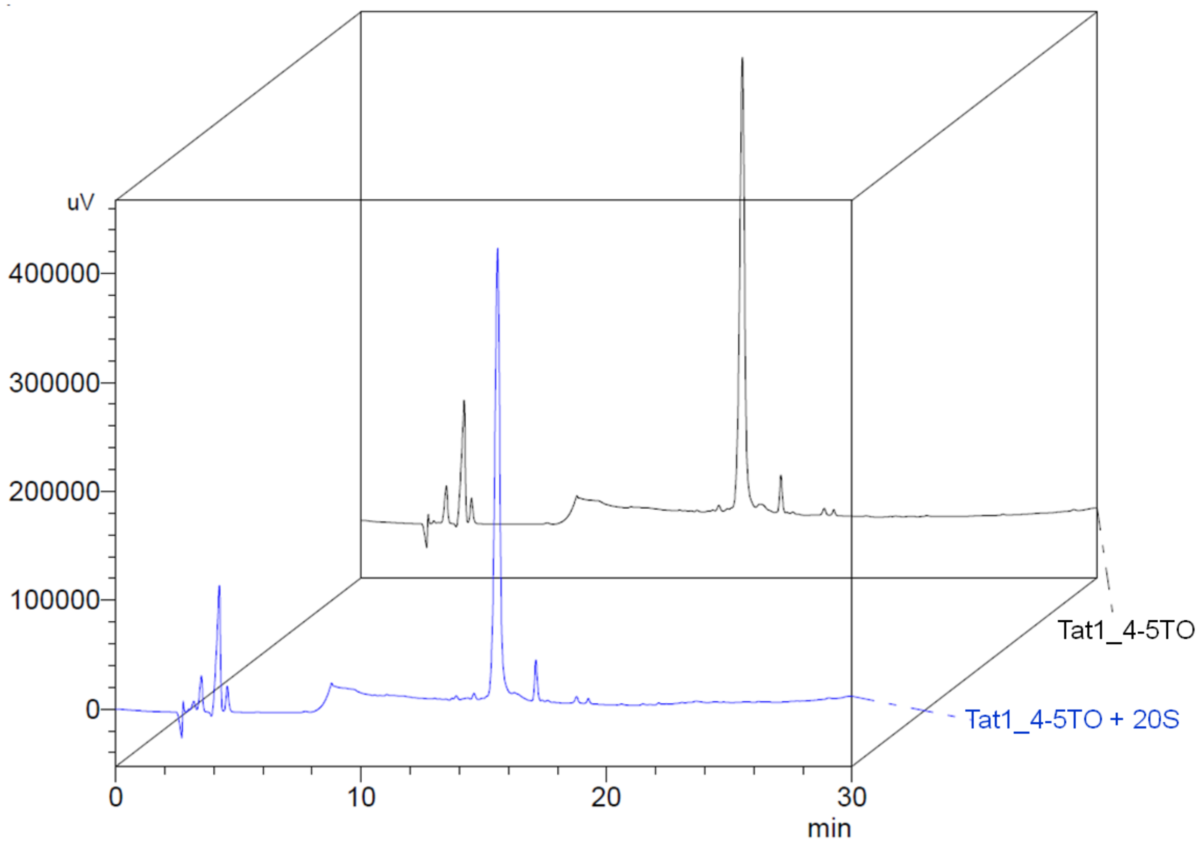

Tat1_8-9TO:

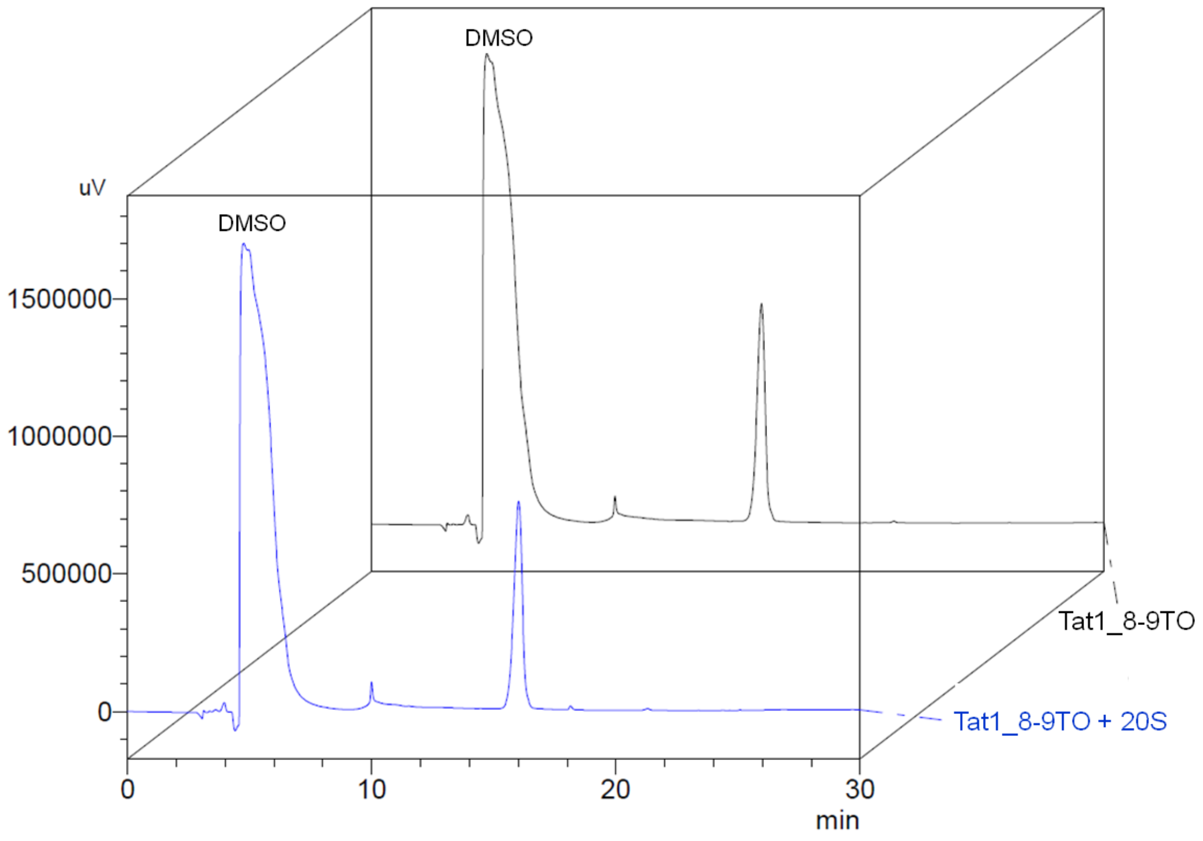

Tat1_8-9TOD:

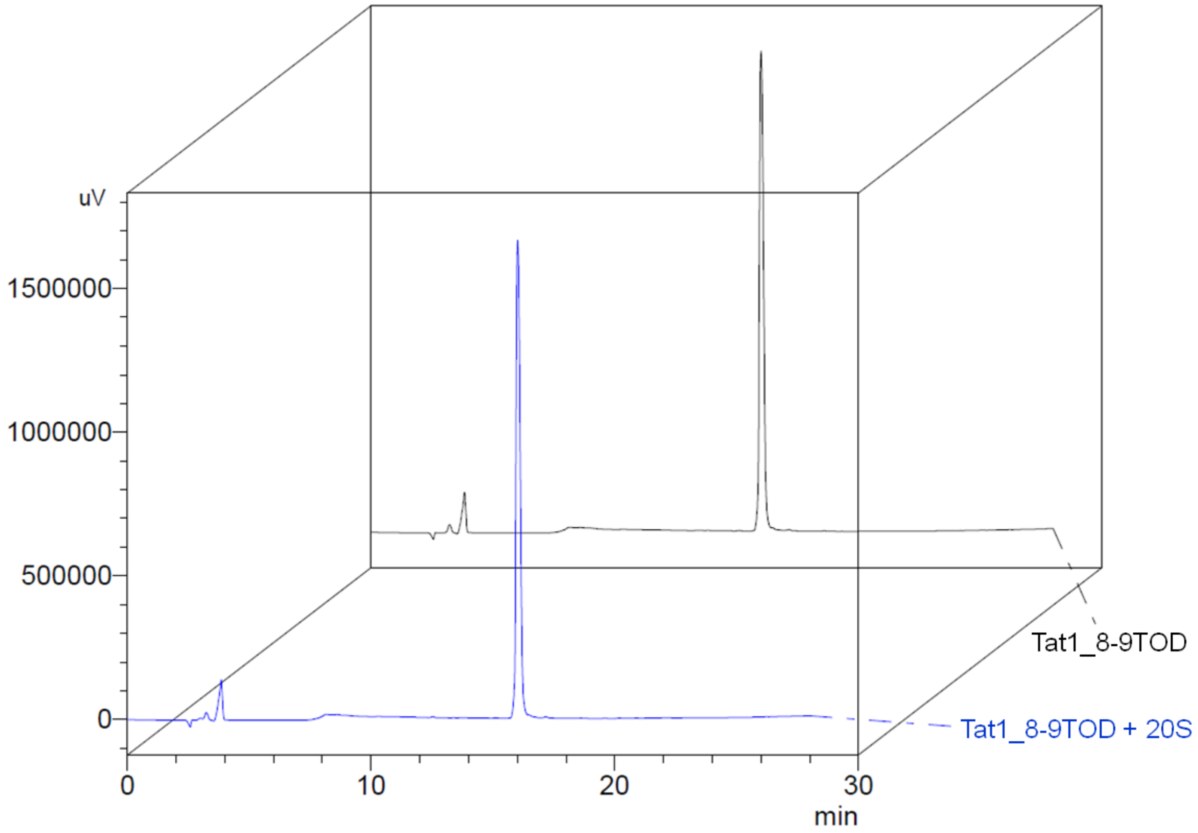

Tat1_4-5TO,8-9TOD:

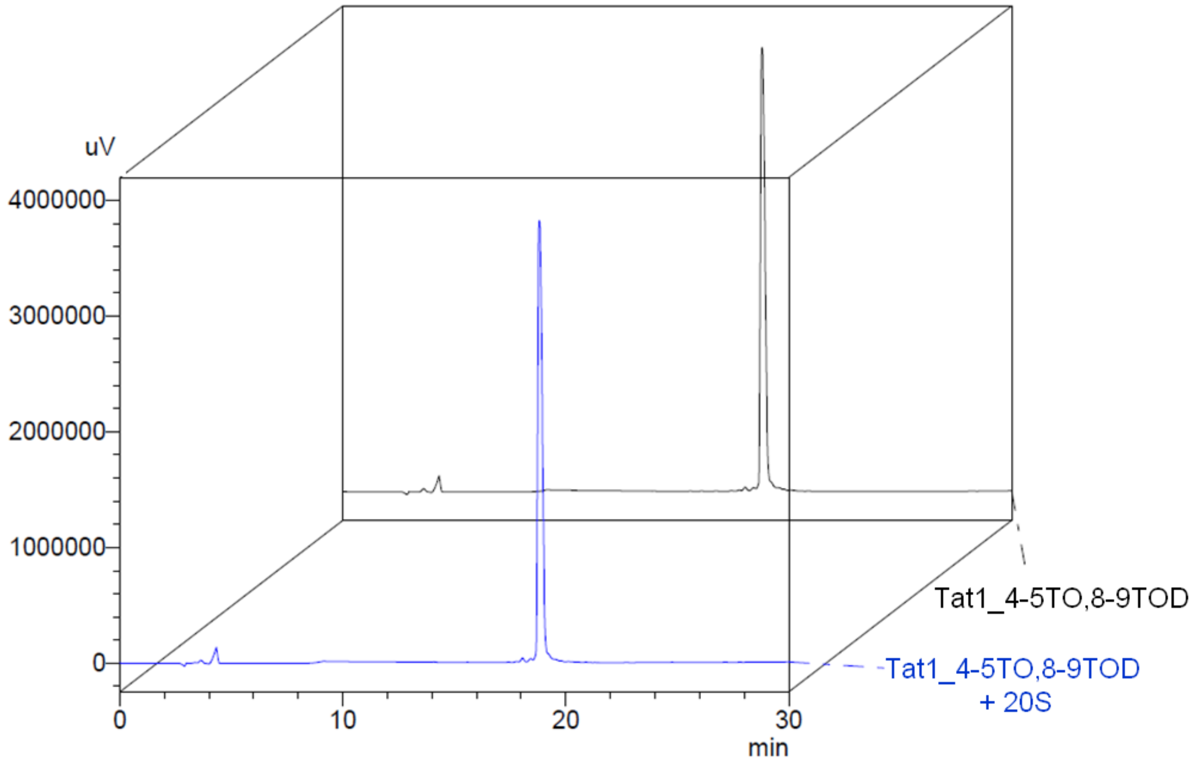

Tat1_A4-5,8-9:

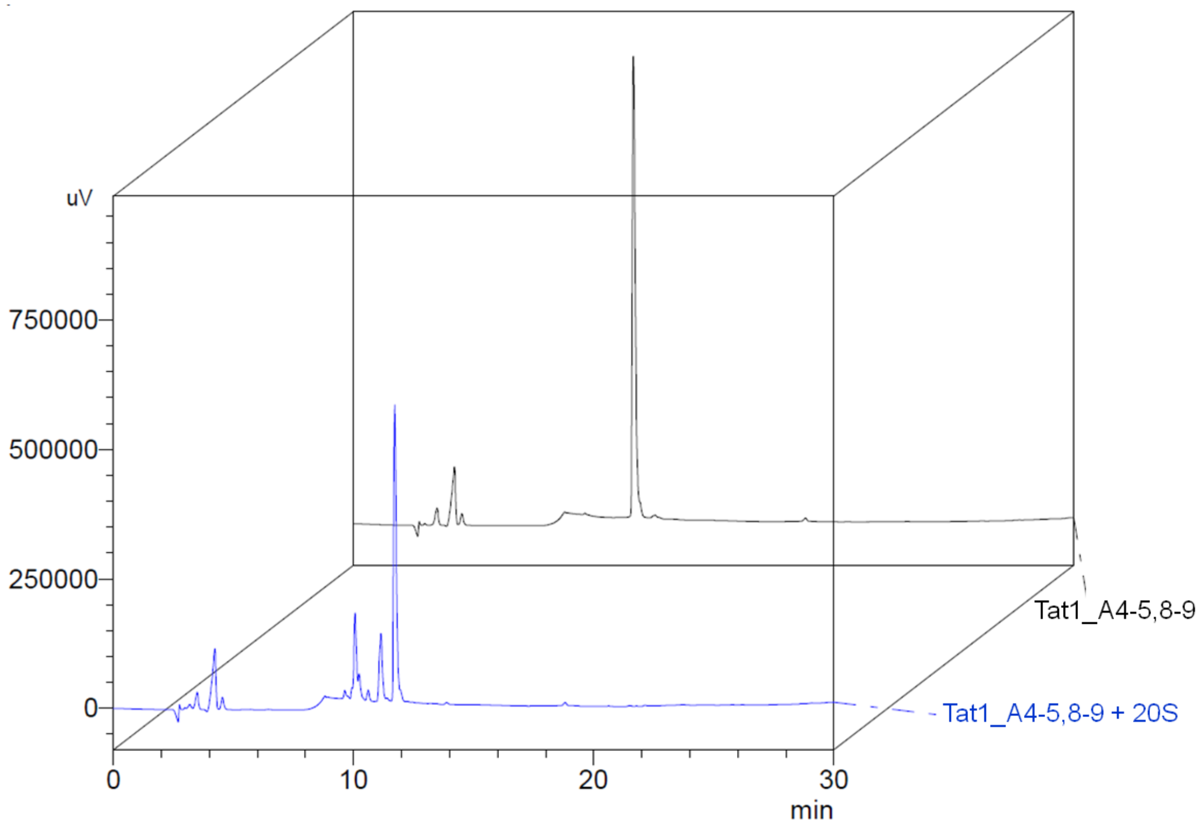
uV
750000
500000
250000
0
0
10
20
30
min
Tat1_A4-5,8-9
Tat1_A4-5,8-9 + 20S
