# Supplementary material for: Interplay between Structure and Charge as a Key to Allosteric Modulation of Human 20S Proteasome by the Basic Fragment of HIV-1 Tat Protein
Source: PLoS One. 2015 Nov 17;10(11):e0143038. doi: 10.1371/journal.pone.0143038 (PMC4648528; doi:10.1371/journal.pone.0143038)
Supplement: S1 Table — (PDF) [file pone.0143038.s013.pdf]

S1 Table. The synthesized analogs of Tat1 with multiple amino acid residues exchanged into Ala or Tic-Oic moiety.

Compound	Sequence	m/z	
		calculated	obtained
Tat1	H-GRKKRRQRRRPS-OH	1580.87	1581.20
Tat1_A3-4	H-GRAARRQRRRPS-OH	1466.68	1466.98
Tat1_A4-5	H-GRKAARQRRRPS-OH	1438.66	1438.35
Tat1_A5-6	H-GRKKAAQRRRPS-OH	1409.84	1409.91
Tat1_A2-4	H-GAAARRQRRRPS-OH	1381.56	1382.42
Tat1_A3-5	H-GRAAARQRRRPS-OH	1381.57	1383.19
Tat1_A4-6	H-GRKAAAQRRRPS-OH	1353.55	1354.00
Tat1_A5-7	H-GRKKAAARRRPS-OH	1353.59	1354.70
Tat1_A5,7-9	H-GRKKARAAARPS-OH	1268.49	1268.52
Tat1_A6-8	H-GRKKRAAARRPS-OH	1353.60	1353.91
Tat1_A7-9	H-GRKKRRAAARPS-OH	1352.82	1352.67
Tat1_A8-10	H-GRKKRRQAAAPS-OH	1325.54	1326.07
Tat1_A7-10	H-GRKKRRAAAAPS-OH	1268.49	1269.79
Tat1_A3-6	H-GRAAAAQRRRPS-OH	1296.46	1296.47
Tat1_A4-5,8-9	H-GRKAARQAARPS-OH	1268.44	1269.30
Tat1_4-5TO	H-GRK-*D*-TicOicRQRRRPS-OH	1607.01	1607.50
Tat1_8-9TO	H-GRKKRRQ-*D*-TicOicRPS-OH	1578.99	1579.78
Tat1_8-9TOD	H-GRKKRRQTic-*D*-OicRPS-OH	1578.99	1579.81
Tat1_4-5TO,8-9TOD	H-GRK-*D*-TicOicRQTic-*D*-OicRPS-OH	1605.13	1605.78
