# Supplementary material for: Interplay between Structure and Charge as a Key to Allosteric Modulation of Human 20S Proteasome by the Basic Fragment of HIV-1 Tat Protein
Source: PLoS One. 2015 Nov 17;10(11):e0143038. doi: 10.1371/journal.pone.0143038 (PMC4648528; doi:10.1371/journal.pone.0143038)
Supplement: S2 Table — (PDF) [file pone.0143038.s014.pdf]

S2 Table. Dissociation constants K_d calculated on the basis of the thermophoresis curves.

**Compound**	**K_d [µM]**
Tat1	4.82 (±0.39)
Tat1_A4-6	12.07 (±1.27)
Tat1_A8-10	13.47 (±0.58)
Tat1_A4-5,8-9	22.27 (±1.41)
Tat1_4-5TO	8.91 (±0.31)
Tat1_8-9TO	10.39 (±1.36)
Tat1_8-9TOD	8.14 (±0.53)
Tat1_4-5TO,8-9TOD	8.38 (±1.16)
