# Supplementary material for: Interplay between Structure and Charge as a Key to Allosteric Modulation of Human 20S Proteasome by the Basic Fragment of HIV-1 Tat Protein
Source: PLoS One. 2015 Nov 17;10(11):e0143038. doi: 10.1371/journal.pone.0143038 (PMC4648528; doi:10.1371/journal.pone.0143038)
Supplement: S3 Table — (PDF) [file pone.0143038.s015.pdf]

**S3 Table.** Proton chemical shifts and the vicinal coupling constants of Tat1_A4-5,8-9 in $H_2O/D_2O$ (9:1) at 298 K.

| Residue | Proton chemical shifts [ppm] | | | | | | $^3J_{HNH\alpha}$ |
|---|---|---|---|---|---|---|---|
| | HN | Hα | Hβ | Hγ | Hδ | others | |
| Gly[1] | | 3.83 | | | | | |
| Arg[2] | 8.70 | 4.32 | 1.73;1.80 | 1.62 | 3.19 | ε-NH 7.21 | 7.2 |
| Lys[3] | 8.64 | 4.27 | 1.73;1.79 | 1.44 | 1.62 | Hε 2.98; ζ-$NH_2$ 7.53 | 7.5 |
| Ala[4] | 8.51 | 4.27 | 1.36 | | | | |
| Ala[5] | 8.45 | 4.26 | 1.36 | | | | |
| Arg[6] | 8.45 | 4.26 | 1.74;1.80 | 1.61 | 3.18 | ε-NH 7.21 | |
| Gln[7] | 8.57 | 4.29 | 1.96;2.06 | 2.36 | | ε-$NH_2$ 6.95;7.64 | 7.7 |
| Ala[8] | 8.53 | 4.29 | 1.37 | | | | |
| Ala[9] | 8.44 | 4.26 | 1.36 | | | | |
| Arg[10] | 8.47 | 4.60 | 1.71;1.84 | 1.68 | 3.21 | ε-NH 7.22 | 7.9 |
| Pro[11] | - | 4.47 | 1.96;2.32 | 2.00 | 3.63;3.82 | | - |
| Ser[12] | 8.60 | 4.47 | 3.88;3.98 | | | | 8.1 |
| | 8.67 | 4.45 | 3.92;3.99 | | | | 8.8 |
| | 8.49 | 4.50 | 3.86;3.93 | | | | |
