# Supplementary material for: Interplay between Structure and Charge as a Key to Allosteric Modulation of Human 20S Proteasome by the Basic Fragment of HIV-1 Tat Protein
Source: PLoS One. 2015 Nov 17;10(11):e0143038. doi: 10.1371/journal.pone.0143038 (PMC4648528; doi:10.1371/journal.pone.0143038)
Supplement: S4 Table — (PDF) [file pone.0143038.s016.pdf]

S4 Table. Proton chemical shifts and the vicinal coupling constants of Tat1_4-5TO in phosphate buffer pH 7.4 at 301 K.

Residue	Proton chemical shifts [ppm] HN	Hα	Hβ	Hγ	Hδ	others	$^{3}J_{HNH\alpha}$
Gly[1]		3.96					
Arg[2]	8.70	4.45	1.80	1.67	3.20	ε-NH 7.12	
Lys[3]	8.50	5.18	1.73	1.43	1.69	Hε 2.94	9.2
	8.66	5.06	1.76	1.49		Hε 3.03	8.9
D-Tic[4]	-	5.27	3.11,3.31			H_5 7.33; H_6 7.25; H_7 7.24; H_8 7.20;	-
		5.60	3.25,3.40				
		5.16	3.11, 3.26				
Oic[5]	-	4.41	2.04,2.27	2.55	4.13	H_4 1.84, 1.36; H_5 1.33,1.54; H_6 1.22,1.75; H_7 1.51, 2.14	-
				2.58	4.21		
					4.12		
Arg[6]	8.05	4.27	1.79,1.8	1.66	3.20	ε-NH 7.20	8.9
Gln[7]	8.24	4.33	1.96,2.04	2.34		ε-NH_2 6.90, 7.55	8.8
	8.55	4.45	2.06,2.13	2.43		*ε-NH_2 6.93,7.59	8.3
	8.29	4.37	2.03	2.39			7.6
Arg[8]	8.37	4.37	1.66,1.78	1.88	3.24	ε-NH 7.23	8.2
Arg[9]	8.42	4.37	1.82	1.66	3.23	ε-NH 7.23	8.4
Arg[10]	8.47	4.67	1.81,1.91	1.66	3.23	ε-NH 7.23	
Pro[11]	-	4.51	2.36,2.05	2.11	3.72,3.88		-
		4.69	2.00,2.44	2.26	3.56,3.67		
Ser[12]	7.98	4.35	3.89				8.8
	7.98	4.29	3.90				8.1
	8.10	4.24	3.93				7.8

*Due to no ROE interactions, it is impossible to correlate these protons with the particular minor conformation of Gln.
