# Supplementary material for: Interplay between Structure and Charge as a Key to Allosteric Modulation of Human 20S Proteasome by the Basic Fragment of HIV-1 Tat Protein
Source: PLoS One. 2015 Nov 17;10(11):e0143038. doi: 10.1371/journal.pone.0143038 (PMC4648528; doi:10.1371/journal.pone.0143038)
Supplement: S5 Table — (PDF) [file pone.0143038.s017.pdf]

S5 Table. Proton chemical shifts and the vicinal coupling constants of Tat1_8-9TO in phosphate buffer pH 7.4 at 301 K.

Residue	Proton chemical shifts [ppm] HN	Hα	Hβ	Hγ	Hδ	others	$^3J_{HNH\alpha}$
Gly[1]		3.94					
Arg[2]	8.65	4.42	1.82,1.88	1.71	3.27	ε-NH 7.24	8.7
Lys[3]	8.52	4.37	1.83	1.51	1.76	Hε 3.07; ζ-NH_2 6.99	7.6
Lys[4]	8.46	4.36		1.53		Hε 3.07; ζ-NH_2 6.99	
Arg[5]	8.45	4.37			3.27		
Arg[6]	8.47	4.37	1.73,1.83	1.92	3.28	ε-NH 7.25	
Gln[7]	8.55	5.18	1.91,2.06	2.36		ε-NH_2 6.87;7.51	9.2
	8.63	4.53	1.90,2.02	2.36		*ε-NH_2 6.87;7.58	8.0
	8.07	4.20	1.99,2.14	2.35		*ε-NH_2 6.94;7.56	8.0
	8.62	5.08	2.04,1.88	2.45			7.6
D-Tic[8]	-	5.22	3.09,3.31			H_5 7.33; H_6 7.36; H_7 7.36; H_8 7.30;	-
		5.65	3.24,3.41				
		5.30	3.10,3.28				
Oic[9]	-	4.42	2.05,2.29	2.53	4.13	H_5 1.35, 1.54; H_6 1.23, 1.75; H_7 1.47, 2.18	-
				2.58	4.19		
				2.57	4.10		
Arg[10]	7.96	4.64	1.85, 1.92	1.73	3.23	ε-NH 7.24	7.8
Pro[11]	-	4.50	2.33	2.06	3.74,3.91		-
		4.53	2.37	2.09	3.72,3.87		
Ser[12]	7.94	4.27	3.87				7.2
	8.05	4.31	3.91				7.7
	8.01	4.30	3.89				8.0
	7.74	4.31	3.87				7.9

*Due to no ROE interactions, it is impossible to correlate these protons with the particular minor conformation of Gln.
