# Supplementary material for: Interplay between Structure and Charge as a Key to Allosteric Modulation of Human 20S Proteasome by the Basic Fragment of HIV-1 Tat Protein
Source: PLoS One. 2015 Nov 17;10(11):e0143038. doi: 10.1371/journal.pone.0143038 (PMC4648528; doi:10.1371/journal.pone.0143038)
Supplement: S6 Table — (PDF) [file pone.0143038.s018.pdf]

S6 Table. Proton chemical shifts and the vicinal coupling constants of Tat1_8-9TOD in phosphate buffer with the reduced pH (pH 4.5) at 301 K.

Residue	Proton chemical shifts [ppm] HN	Hα	Hβ	Hγ	Hδ	others	$^3J_{HNH\alpha}$
Gly[1]		3.91					
Arg[2]	8.64	4.39	1.79,1.85	1.68	3.24	ε-NH 7.21	6.9
Lys[3]	8.51	4.33	1.79	1.47	1.71	Hε 3.03; ζ-NH_2 7.55	6.9
Lys[4]	8.44	4.33	1.78,1.82	1.45	1.65	Hε 3.03	
Arg[5]	8.44	4.25			3.23	ε-NH 7.09	
Arg[6]	8.44	4.25			3.23	ε-NH 7.09	
Gln[7]	8.66	5.03	1.97,2.13	2.41		ε-NH_2 6.95, 7.57	8.4
	8.57	5.04	1.98,2.08	2.33			8.4
	8.68	5.02	1.96,2.12	2.42			8.1
Tic[8]	-	5.12	3.09,3.16		4.67,4.94	H_5 7.30; H_6 7.33; H_7 7.33; H_8 7.27;	-
		5.07	3.17,3.10				
		4.25	2.96		4.62,5.00	H_5 7.21	
D-Oic[9]	-	4.41	2.52	2.52	4.16	H_4 1.49,1.77; H_5 1.33, 1.48; H_6 1.19,1.75; H_7 1.41,1.99	-
					4.17		
		5.01	2.73	2.43	4.08		
Arg[10]	7.78	4.71	1.85	1.61	3.16	ε-NH 7.15	8.5
	7.73	4.73	1.85	1.63	3.16		
	7.71	4.72	1.85	1.64	3.19		
Pro[11]	-	4.40	2.26	1.90,1.99	3.62,3.69		-
		4.43	2.40	2.08	3.74,3.95		
		4.46	2.26		3.63,3.70		
Ser[12]	8.00	4.26	3.86				7.1
	7.85	4.21	3.84				7.7
	7.97	4.30	3.85				8.3
	8.09	4.19	3.89				
