# Supplementary material for: Interplay between Structure and Charge as a Key to Allosteric Modulation of Human 20S Proteasome by the Basic Fragment of HIV-1 Tat Protein
Source: PLoS One. 2015 Nov 17;10(11):e0143038. doi: 10.1371/journal.pone.0143038 (PMC4648528; doi:10.1371/journal.pone.0143038)
Supplement: S7 Table — (PDF) [file pone.0143038.s019.pdf]

S7 Table. Proton chemical shifts and the vicinal coupling constants of Tat1_4-5TO,8-9TOD in phosphate buffer with the reduced pH (pH 4.5) at 301 K.

Residue	Proton chemical shifts [ppm]**						$^{3}J_{HNH\alpha}$
	HN	Hα	Hβ	Hγ	Hδ	others	
Gly[1]		3.89					
Arg[2]	8.68	4.41	1.77	1.59	3.05	ε-NH 6.97	9.3
Lys[3]	8.52	5.17	1.71	1.43	1.66	Hε 2.93	8.5
	8.47	5.23	1.79	1.46	1.71	Hε 2.95	
D-Tic[4]	-	5.23	3.10,3.29		4.68,4.93	H_5 7.31	-
Oic[5]	-	4.35	1.97,2.26	2.53	4.11	H_6 1.22,1.74; H_7 1.46,2.11	-
Arg[6]	7.98	4.18	1.58,1.68	1.46	3.07	ε-NH 7.10	
Gln[7]	8.42	5.00	1.97,2.10	2.34		ε-NH_2 6.91,7.53	
	8.32	4.96	2.00,2.23	2.44		*ε-NH_2 6.84,7.46	
	8.38	4.99	2.02	2.23		*ε-NH_2 6.94,7.60	
	8.64	5.10	2.03	2.41		*ε-NH_2 6.97,7.57	
Tic[8]	-	5.15	3.14,3.33		4.62,4.80	H_5 7.22	-
		4.24	2.96			H_5 7.21	
D-Oic[9]	-	4.42	2.24,2.52	2.55	4.16	H_6 1.22,1.76; H_7 1.46,2.04	-
		5.00	2.74		4.07		
Arg[10]	7.82	4.69	1.67,1.85	1.61	3.18	ε-NH 7.18	8.4
	8.70	4.74	1.77,1.97	1.62	3.28	ε-NH 7.29	7.1
	7.79	4.69	1.68,1.86	1.62	3.17	ε-NH 7.18	8.4
Pro[11]	-	4.40	2.26	1.89,2.00	3.64,3.70		-
		4.56	2.44	2.10	3.95		
Ser[12]	7.97	4.27	3.87				6.5
	7.86	4.24	3.86				
	8.03	4.27	3.89				

*Due to no ROE interactions, it is impossible to correlate these protons with the particular minor conformation of Gln.

**In addition to the data presented in the table, there were signals difficult to unambiguous assignment. Indeed, it is possible to recognize a spin system and consequently amino acid residue (Arg and Tic), however due to no interesidue ROE cross peaks, we cannot confirm the position of residue in the sequence.
